# The effect of hearing ability on dual-task performance following multi-domain training in older adults with mild cognitive impairment: findings from the SYNERGIC trial

**DOI:** 10.3389/fnagi.2025.1716733

**Published:** 2026-01-30

**Authors:** Rachel I. Downey, Berkley J. Petersen, Niroshica Mohanathas, Jennifer L. Campos, Manuel Montero-Odasso, Louis Bherer, M. Kathleen Pichora-Fuller, Nick W. Bray, Amer M. Burhan, Richard Camicioli, Sarah Fraser, Teresa Liu-Ambrose, Maxime Lussier, Laura E. Middleton, Frederico Pieruccini-Faria, Natalie A. Phillips, Karen Z. H. Li

**Affiliations:** 1 Department of Psychology/Centre for Research in Human Development, Concordia University, Montréal, QC, Canada; 2 School of Health, Concordia University, Montréal, QC, Canada; 3 Department of Psychology, University of Toronto, Toronto, ON, Canada; 4 KITE-Toronto Rehabilitation Institute, University Health Network, Toronto, ON, Canada; 5 Gait and Brain Lab, Parkwood Institute, Lawson Health Research Institute, London, ON, Canada; 6 Department of Medicine, Division of Geriatric Medicine, Schulich School of Medicine and Dentistry, University of Western Ontario, London, ON, Canada; 7 Department of Epidemiology and Biostatistics, Schulich School of Medicine & Dentistry, Western University, London, ON, Canada; 8 Département de Médecine, Université de Montréal, Montréal, QC, Canada; 9 Centre de Recherche de l’Institut de Cardiologie de Montréal, Montréal, QC, Canada; 10 Centre de Recherche de l’Institut Universitaire de Gériatrie de Montréal, Montréal, QC, Canada; 11 Recovery and Performance Lab, Division of Biomedical Sciences, Faculty of Medicine, Memorial University of Newfoundland, St. John's, NL, Canada; 12 Ontario Shores Centre for Mental Health Sciences, Whitby, ON, Canada; 13 Division of Geriatric Psychiatry, Department of Psychiatry, Temerty Faculty of Medicine, University of Toronto, Toronto, ON, Canada; 14 Department of Medicine (Neurology), University of Alberta, Edmonton, AB, Canada; 15 Neuroscience and Mental Health Institute, University of Alberta, Edmonton, AB, Canada; 16 Interdisciplinary School of Health Sciences, Faculty of Health Sciences, University of Ottawa, Ottawa, ON, Canada; 17 The Centre for Aging SMART, Vancouver Coastal Health Research Institute, Vancouver, BC, Canada; 18 Djavad Mowafaghian Centre for Brain Health, Vancouver Coastal Health Research Institute, Vancouver, BC, Canada; 19 Department of Kinesiology and Health Sciences, University of Waterloo, ON, Canada; 20 Schlegel-UW Research Institute for Aging, Waterloo, ON, Canada

**Keywords:** cognitive training, dual-task, exercise, gait, hearing loss, mild cognitive impairment, multi-domain training

## Abstract

**Background:**

Hearing loss is one of the largest potentially modifiable risk factors for dementia and is linked with poor cognitive-motor dual-task performance (e.g., walking while performing a cognitive task). Hearing loss is more prevalent and severe in males, whereas dementia is more prevalent in females. Physical exercise and cognitive interventions appear promising in improving dual-tasking in older adults; however, it is currently unclear whether hearing ability affects training efficacy on dual-task outcomes in older adults with mild cognitive impairment (MCI), and whether sex influences this effect.

**Objective:**

The primary aim of this study was to examine whether hearing ability affects dual-task performance at baseline and after training in individuals with MCI, and whether sex further influences these relationships, irrespective of intervention arm.

**Methods:**

Secondary data was analysed from 75 participants with MCI (*M_age_* = 73.66 ± 6.67) enrolled in the SYNERGIC trial. Hearing ability was assessed using self-report and behavioral measures. Participants completed a 20-week intervention: (1) Exercise (aerobic-resistance exercise + sham cognitive training; *n* = 31), (2) Multi-Domain Training (aerobic-resistance exercise + cognitive training; *n* = 32), or (3) Placebo Training (balance and toning exercises + sham cognitive training; *n* = 12). Primary outcomes included dual-task gait and cognitive performance.

**Results:**

At baseline, poorer hearing predicted worse dual-task performance, particularly in males. Dual-task gait variability significantly improved following Multi-Domain Training in participants with a greater degree of self-reported hearing complaints. Sex-stratified analyses revealed that females with more hearing complaints improved more across all interventions, while in the Multi-Domain group, males with poorer objective hearing and females with better hearing showed the greatest gains. Additionally, in those with poorer hearing, lower cognitive scores (MoCA) predicted greater improvements after Multi-Domain Training, but a decline after Placebo Training.

**Conclusion:**

Hearing ability, sex, and cognitive status appear to interact to influence the effects of exercise and cognitive training on dual-task performance in older adults with MCI. Multi-Domain Training appears particularly beneficial for those with hearing loss (who are male and/or have lower cognitive status), highlighting the need for personalized interventions to preserve function and slow decline in this at-risk population.

**Clinical trial registration:**

https://www.clinicaltrials.gov/ct2/show/NCT02808676, NCT02808676.

## Introduction

Between 16 and 22% of older adults over the age of 65 have mild cognitive impairment (MCI; Manly et al., 2022; Roberts and Knopman, 2013). MCI is considered a transitional state between age-normative cognitive functioning and dementia, with annual conversion rates of MCI to dementia ranging between 10 and 15% (Petersen, 2004; Roberts and Knopman, 2013). While research demonstrates that females have a greater risk of developing dementia compared to males (Cao et al., 2020), there is mixed evidence for sex differences in MCI (e.g., a meta-analysis found no sex differences in amnestic MCI, but found a higher prevalence of non-amnestic MCI in females compared to males; Au et al., 2017). In addition to cognitive impairment, individuals with MCI also commonly experience deficits in gait and balance (Bahureksa et al., 2016) and are at an increased risk for falls (Liu-Ambrose et al., 2008). Cognitive-motor dual-tasking (i.e., simultaneous completion of a cognitive and motor task) is a sensitive marker of cognitive and physical decline in older adults (Beauchet et al., 2009; Ramírez and Gutiérrez, 2021). Indeed, studies have shown that older adults with MCI have slower and more variable gait during dual-tasking, compared to cognitively unimpaired older adults (Montero-Odasso et al., 2012a; Muir et al., 2012). Targeting this early prodromal stage of dementia is widely considered the optimal stage for intervention to slow the progression of decline (Petersen, 2004).

Non-pharmacological interventions have been shown to improve cognitive functioning in cognitively unimpaired older adults, as well as older adults with MCI or dementia, including aerobic exercise and resistance training (Falck et al., 2019; Huang et al., 2022) and computerized cognitive training (Chan et al., 2024; Hill et al., 2017; Zhang et al., 2019). There is also evidence that physical exercise and executive function training can improve cognitive-motor dual-task performance in cognitively unimpaired older adults, such as dual-task auditory working memory performance (Downey et al., 2022), postural control (Fraser et al., 2017; Li et al., 2010; Smith-Ray et al., 2015) and gait (Fraser et al., 2017; Marusic et al., 2018; Plummer et al., 2015). There is currently mixed evidence for the potential synergistic effect of combined physical exercise and computerized cognitive training, with some studies demonstrating improvements to global cognition (Montero-Odasso et al., 2023), gait speed (Pieruccini-Faria et al., 2025) and cognitive dual-task performance (Bherer et al., 2021), while others show no synergistic benefit (e.g., Desjardins-Crépeau et al., 2016; Fraser et al., 2017). As such, further research into the potential synergistic benefits of physical exercise and cognitive training on dual-task performance in older adults with MCI is needed.

In addition to cognitive and physical decline, hearing loss is also highly prevalent in late adulthood, affecting up to two thirds of older adults over the age of 70 (Lin et al., 2011). Sex differences have also been found in age-related hearing loss, with hearing loss being more common, more severe, and having an earlier onset in males compared to females (Nolan, 2020). Hearing loss has been identified as one of the largest potentially modifiable risk factors for dementia (Livingston et al., 2017, 2020, 2024). Additionally, cognitive-motor dual-task performance has been found to differ across participants with normal hearing and hearing loss. Specifically, compared to older adults with normal hearing, older adults with hearing loss have exhibited reduced auditory working memory performance when simultaneously engaged in a postural control task (Bruce et al., 2019a), and higher dual-task costs in gait speed and cadence (Wollesen et al., 2018). Hearing loss is also associated with postural instability and an increased risk of falling in older adults (Agmon et al., 2017; Campos et al., 2018; Carpenter and Campos, 2020; Foster et al., 2022). Most prior research examining associations between hearing and cognitive or mobility outcomes has relied primarily on pure-tone audiometry, with comparatively less attention to central auditory processing measures or self-reported hearing difficulties, warranting further investigation using complementary approaches.

There is some evidence that hearing aid use may reduce cognitive decline over time, particularly in older adults who have poorer baseline cognitive functioning (Lin et al., 2023). However, the effect of hearing aids on mobility is mixed, with variable results for static and dynamic balance outcomes (Borsetto et al., 2021). Other interventions, such as cognitive training and auditory training (i.e., refining the sensory perception of sounds to improve speech perception), have also been shown to improve cognitive functioning in cognitively unimpaired older adults with hearing loss (Lawrence et al., 2018). Nevertheless, to our knowledge, there has been no previous research exploring the effect of cognitive and physical training interventions on cognition and mobility in older adults with comorbid hearing loss and MCI.

According to compensatory theories of cognitive aging, physical exercise and cognitive training may enhance neural scaffolding, or the engagement/upregulation of supplementary neural activity to counteract age-related neurofunctional decline (Reuter-Lorenz and Park, 2014). Li et al. (2018) further proposed that compensatory scaffolding not only assists in preserving cognitive functioning in the aging brain, but also sensory and motor functioning, such as cognitive-motor dual-tasking. It has been postulated that in individuals with hearing loss, auditory deprivation over time leads to structural and functional brain changes, such as temporal lobe atrophy and the re-allocation of cognitive resources to support auditory processing (Lin et al., 2014; Peelle and Wingfield, 2016; Powell et al., 2022). As such, physical exercise and cognitive training may enhance compensatory scaffolding, and in turn, dual-task performance, and these benefits may extend to individuals with MCI and poorer hearing who may have alterations in brain structure and function caused by reduced auditory processing over time.

Taken together, there is moderate evidence for the efficacy of physical exercise and cognitive interventions to improve dual-task performance in older adults, with mixed evidence for a potential synergistic effect of combined physical exercise and cognitive training. However, there have yet to be any investigations into the effects of hearing ability on dual-task outcomes in older adults with MCI before and after physical exercise and cognitive training. Additionally, while there are known sex differences in the prevalence rates of dementia and hearing loss in older adults, it is currently unknown whether biological sex influences the relationship between hearing loss severity and dual-task performance in older adults with MCI before and after training.

### Research objectives and hypotheses

By utilizing a subset of data from the SYNnchronizing Exercises, Remedies in Gait and Cognition (SYNERGIC) trial (Identifier: NCT02808676; i.e., including participants who were co-enrolled in a longitudinal study that included a hearing evaluation), the primary objectives of this study were to determine whether hearing ability predicts cognitive-motor dual-task performance at baseline, and if degree of hearing loss predicts training-related improvements in dual-task performance following physical exercise, alone or in combination with cognitive training. Both self-reported and objective measures of hearing were included because they capture related but distinct constructs, with self-report reflecting perceived hearing difficulties in everyday contexts and objective testing indexing auditory processing under controlled conditions. We were also interested in exploring whether any sex differences would emerge for each research objective. Of note, while the intervention arms were originally designed to test potential synergistic effects of combined physical exercise and cognitive training, the present set of analyses examine hearing-related effects within each arm rather than direct arm-to-arm comparisons.

We hypothesized that with increasing severity of hearing loss, there would be poorer cognitive and gait dual-task performance at baseline, and a higher magnitude of improvement in dual-task performance following Multi-Domain Training and Exercise Training, with the greatest magnitude of improvement following Multi-Domain Training. Additionally, given that hearing loss tends to be more severe and have an earlier age of onset in males, we further hypothesized that the relationship between hearing ability and dual-task performance would be stronger in males compared to females at baseline, and that males with a greater degree of hearing loss would improve more following training. Specifically, we posited that males with hearing loss may have greater alterations in brain structure and function (e.g., greater temporal lobe atrophy, increased reliance on frontal compensatory networks; Powell et al., 2022), which would increase demands at baseline, leading to reduced performance, though would allow for increased uptake and a greater opportunity for improvement following cognitive or physical training.

## Methods

### Participants

Participants were comprised of a subset of individuals from the SYNERGIC trial who were co-enrolled in a longitudinal study that included a hearing evaluation (i.e., Comprehensive Assessment of Neurodegeneration and Dementia; COMPASS-ND), which was carried out under the auspices of the Canadian Consortium on Neurodegeneration and Aging (CCNA). Participants were included in the current analyses if they were between 60 and 85 years old and had a diagnosis of MCI (i.e., cognitive impairment in one of the following four cognitive domains: memory, executive function, attention, and language, with preserved activities of daily living and absence of dementia). Participants were excluded if they had an uncontrolled psychiatric disorder, a neurological disorder with residual motor deficits, uncontrolled hypertension, diabetes, known renal/kidney insufficiency, or were actively participating in another physical exercise program and/or clinical trial. There was no exclusion criterion based upon hearing ability (i.e., participants were not excluded if they were diagnosed with a hearing impairment or if they wore hearing aids). Full details concerning recruitment and eligibility are described elsewhere (Chertkow et al., 2019; Montero-Odasso et al., 2018, 2023).

The SYNERGIC trial aimed to recruit a total of 200 participants (40 per training group) to investigate the effect of physical exercise, alone or in combination with cognitive training, and Vitamin D_3_ supplementation on global cognition (Montero-Odasso et al., 2018, 2023). The sample size calculation for the current set of analyses was based upon results from Fraser et al. (2017), who used a similar physical exercise and executive function training protocol with different combinations of active and control conditions and reported pre- and post-training changes in our primary outcome measures (i.e., dual-task gait, auditory working memory) in cognitively healthy older adults. In order to achieve a power of 0.95 at an alpha level of 0.05 for a main effect of Time (pre- vs. post-training) for cognitive accuracy, the recommended sample size was 30 participants total (i.e., 10 participants per training group).

**Figure 1:**
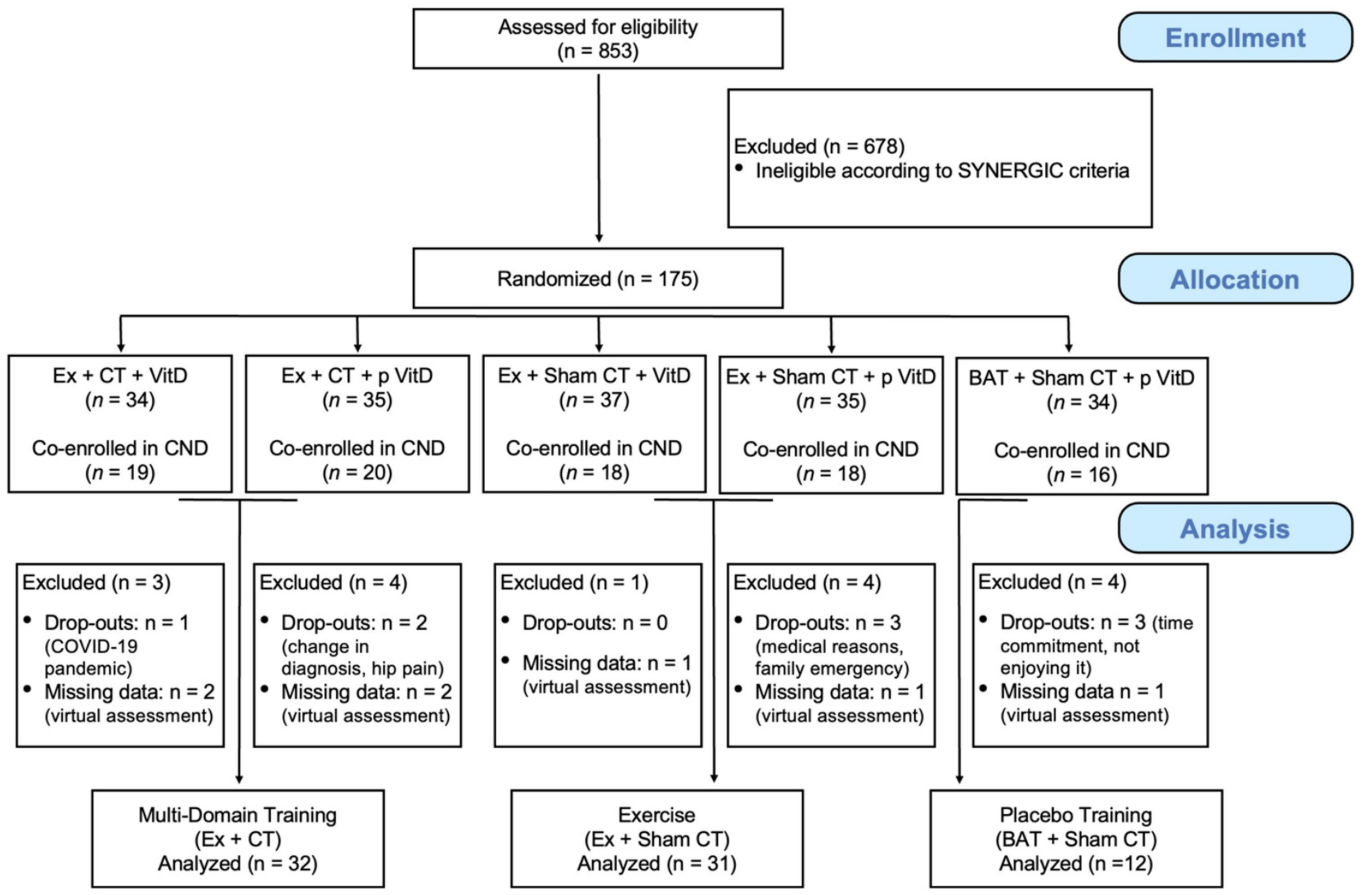
Consort flow diagram of participant recruitment and group allocation. Ex, Aerobic-resistance exercise; CT, Cognitive training; BAT, Balance and toning; VitD, Vitamin D_3_; p, Placebo; CND, COMPASS-ND. Results from Montero-Odasso et al. (2023) and the current set of analyses did not reveal any significant differences between Vitamin D_3_ supplementation versus placebo on our primary outcome measures. We therefore pooled the data across the Vitamin D groups to increase statistical power, leading to a total of three treatment arms.

A total of 853 participants were screened for eligibility to participate in the SYNERGIC study. Following screening procedures, 175 participants were deemed eligible and were randomized to one of five treatment arms (see Interventions section for full description of the intervention arms). Of note, in previous studies involving the same training design and participants as the current study, no significant differences were found between Vitamin D supplementation versus placebo on cognitive performance (Montero-Odasso et al., 2023) or dual-task gait (Pieruccini-Faria et al., 2025). As such, we pooled the data across the Vitamin D groups. Of the 175 participants, 90 were co-enrolled in the COMPASS-ND study. Of these 90 participants, 15 participants were excluded from the analyses (9 withdrew, 6 had missing data due to the COVID-19 pandemic). Therefore, a total of 75 participants (*M_age_* = 73.66 years ± 6.67) were included in the study analyses: 32 in the Multi-Domain Training group, 31 in the Exercise group, and 12 in the Placebo Training group (See Figure 1 for CONSORT flow diagram, and Table 1 for participants characteristics across training groups).

**Table 1:** Means, standard deviations, and one-way ANOVAs/Chi-square tests for participant characteristics across training groups at baseline.

Measure	Multi-domain training *n* = 32		Exercise *n* = 31		Placebo training *n* = 12		*F* or *χ^2^*	*p*	*η^2^*
	*M*	*SD*	*M*	*SD*	*M*	*SD*			
Age (years)	72.59	7.60	74.65	6.24	73.92	5.71	0.730	0.486	0.020
Education (years)	15.34	2.86	15.32	3.87	17.71	6.01	1.86	0.163	0.049
MoCA (/30)	23.97	2.90	22.48	2.72	21.58	3.18	3.78*	0.027	0.095
HHIE-S (/40)	9.63	10.28	7.23	7.71	7.82	8.69	0.576	0.565	0.016
CDTT SRT (SNR)	−8.94	3.72	−9.03	3.13	−8.10	5.85	0.184	0.833	0.007
Sex (*n*, % female)	14 (43.75)		10 (32.26)		8 (66.67)		4.21	0.122	
Hearing aid use (*n*)	5		3		1		1.75	0.417	

Multi-domain training consisted of aerobic-resistance exercise with cognitive training; Exercise consisted of aerobic-resistance exercise with sham cognitive training; Placebo training consisted of balance and toning exercises with sham cognitive training. MoCA, Montreal Cognitive Assessment; HHIE-S, Hearing Handicap Inventory for The Elderly – Screening Version; CDTT SRT, Canadian Digit Triplet Test – Speech Response Threshold; SNR, Signal to noise ratio; *p < 0.05.

### Demographics and background measures

Demographic information was collected during a screening visit for the SYNERGIC trial. In the current study, we included the participants’ self-reported age, sex, years of education, and hearing aid use. Global cognitive functioning was measured with the Montreal Cognitive Assessment (MoCA; total score out of 30; Nasreddine et al., 2005). Hearing ability was assessed in the COMPASS-ND study using a self-report questionnaire and two behavioral measures. Specifically, participants completed the Hearing Handicap Inventory for The Elderly - Screening Version (HHIE-S; total score of 40; Ventry and Weinstein, 1983), a 10-item questionnaire designed to evaluate the emotional and social impacts of hearing loss, with higher scores indicating a greater disability associated with hearing loss. An abbreviated pure-tone audiometry assessment was conducted as a screening procedure. Participants were assigned to one of two hearing loss categories based on their ability to detect 2-kHz pure tones. Supplementary analyses were conducted using this measure to examine the effect of hearing status (defined categorically as impaired or unimpaired) on dual-task performance before and after Multi-Domain Training, Exercise, or Control. The methodology on the abbreviated pure-tone audiometry assessment is included in the Supplementary material. Additionally, the descriptive statistics for the participant characteristics across hearing categories are listed in Supplementary Table 1. The Canadian Digit Triplet Test (CDTT; Giguère et al., 2020) was administered to examine suprathreshold hearing abilities in noise and was the primary behavioral measure of hearing loss included in the study analyses. The test was administered in a quiet office using a Dell laptop, a pair of headphones (DD45), and a response keypad. For each of 24 trials, participants heard a set of three digits presented among background noise. Participants were instructed to enter the digits heard in order on each trial using a touch-tone phone keypad. The level of noise presented on each trial was adjusted using an automated adaptive procedure. The outcome variable of interest was the speech reception threshold (SRT) in noise, which was computed through the CDTT’s adaptive scoring procedure. The SRT corresponds to the signal-to-noise ratio (SNR) at which the triplets are correctly recognized 50% of the time.

### Primary outcome measures

As part of the SYNERGIC trial, single- and dual-task performances were evaluated using three different cognitive tasks involving working memory and updating (i.e., Serial 1 and 7 Subtractions) and Semantic Fluency (i.e., naming animals). For the Serial Subtraction conditions, participants were given a random three-digit number and instructed to continuously subtract either one or seven from their answer out loud. Responses following the starting number were recorded for accuracy. If the participant made an error, subsequent correct subtractions were considered accurate (e.g., if the starting number was 100 and the participant responded 93, 87, 80, they would receive an accuracy score of 2). For the semantic fluency condition, participants were asked to generate as many animals as they could think of out loud. Responses were recorded to obtain a measure of accuracy (i.e., total number of responses minus repetitive and intrusion errors). The single-task cognitive conditions (i.e., Serial 7 Subtractions, Semantic Fluency) were completed while seated and participants were given 10 s to complete each of the cognitive tasks. The dual-task cognitive conditions (Serial 1 And 7 Subtractions, Semantic Fluency) involved the simultaneous completion of the cognitive tasks while walking (more details below). Participants first completed the single-task cognitive trials before the gait and dual-task assessments were completed. The amount of time participants had to complete each cognitive dual-task depended on how quickly the walking task was completed. To account for differences in the time to complete each task, an accuracy divided by time score was calculated for each single- and dual-task condition.

The gait task involved walking at a usual pace along a 6-meter gait mat (ProtoKinetic®, GAITRite® Systems, Inc.). To account for acceleration and deceleration of gait, participants were instructed to start walking 1 m before the gait mat and end walking 1 m beyond the gait mat (Cullen et al., 2018). Single-task walking trials were completed three times. Under dual-task conditions, participants walked along the gait mat once for each of the three cognitive tasks. The gait mat allowed for the measurement of spatiotemporal gait characteristics using embedded electronic pressure sensors. Gait parameters included gait speed (cm/s), stride time (ms; i.e., time elapsed between the first contact of two consecutive footsteps of the same foot), stride length (cm; i.e., distance between successive points of initial contact of the same foot), and double-support time (ms; portion of stride time in which both feet are on the ground). In addition to mean performance calculated across multiple gait cycles, the coefficient of variation (CV = (standard deviation/mean) × 100) was also calculated for each of these measures to quantify gait variability. Additionally, proportional dual-task costs were calculated as follows: ((single-task – dual-task)/single-task)*100.

### Study procedures

The hearing assessments were conducted in an initial screening visit in the COMPASS-ND study, and the dual-task experiment was conducted in the SYNERGIC trial. The mean interval between the COMPASS-ND hearing assessments and the SYNERGIC dual-task assessment was 23 days (*SD* = 234.35), with a small sample of participants (*n* = 4) having completed the hearing assessments more than 1 year prior to the dual-task gait assessment. Participants completed 20 weeks of training, which was followed by the same dual-task assessment post-training. Data were stored and accessed from the Longitudinal Online Research and Imaging System (LORIS) database (Mohaddes et al., 2018). Refer to Chertkow et al. (2019) for the complete COMPASS-ND study protocol, as well as Montero-Odasso et al. (2018, 2023) and Bray et al. (2023a) for the SYNERGIC study protocol. The COMPASS-ND study was approved by the Jewish General Hospital Research Ethics Board. For the SYNERGIC study, all procedures were approved by the Research Ethics Board at the University of Western Ontario (REB# 107670), the Lawson Health Research Institute’s Clinical Research Impact Committee (R-15-038), and Health Canada (HC file-HC6–24-c195918 / HC protocol #201619). Each intervention site also obtained local ethical approval. Finally, study procedures, including retrospective data analyses, were approved by Concordia University’s Human Research Ethics Committee (Certificate #30014926). All participants provided informed consent.

### Interventions

Participants were randomized to one of five treatment arms, involving both active and control interventions, including (1) Aerobic-resistance exercise (Ex) + Cognitive Training (CT) + Vitamin D_3_, (2) Ex + CT + Vitamin D placebo, (3) Ex + Sham CT + Vitamin D_3_, (4) Ex + Sham CT + Vitamin D placebo, and (5) Balance and Toning (BAT) exercises + Sham CT + Vitamin D placebo. As previously noted, results from Montero-Odasso et al. (2023) and the current set of analyses revealed no significant differences between Vitamin D supplementation versus placebo on cognitive and dual-task outcomes. We therefore pooled the data across the Vitamin D groups, leading to a total of three treatment arms: (1) Multi-Domain Training: Ex + CT (2) Exercise: Ex + Sham CT, and (3) Placebo Training: BAT + Sham CT. Interventions were completed in small groups (up to eight participants) three times a week for 20 weeks. Each session lasted approximately 90 min, with 30 min devoted to the cognitive training or cognitive sham program, followed by 60 min of active physical exercise or the placebo BAT exercises (see Montero-Odasso et al., 2018, 2023 for further details).

#### Physical exercise interventions

The active physical exercise intervention involved upper and lower body resistance training, as well as aerobic exercise (e.g., walking, cycle ergometers). Intensity of physical exercise was monitored using the Borg Rating of Perceived Exertion (Borg, 1982), which progressed for the aerobic exercise component throughout the training every 4 weeks. The placebo physical exercise intervention (BAT) was designed to improve muscle tone and flexibility (e.g., stretching, balance, toning exercises), without improving strength or aerobic capacity. The control exercises did not progress in volume or intensity.

#### Cognitive interventions

The cognitive intervention (Neuropeak) was completed on individual tablets (iPad®) and involved dual-task training aimed at improving divided attention (i.e., sharing attention between two simultaneous tasks). The cognitive exercises progressed in difficulty across the 20 weeks of training. Specifically, the first 30 training sessions were performed following a fixed priority instruction (during dual-task trials, participants were asked to keep an equal priority across tasks). The following 30 sessions were performed following a variable priority instruction (during dual-task trials, participants were instructed to prioritize one task over the other, which varied across blocks). In the sham cognitive training condition, participants completed two different tasks (i.e., internet searching and video watching), which alternated across sessions, using the same tablet (iPad®) as the cognitive training group. The duration of time to complete the tasks was the same as the cognitive training.

### Statistical analyses

Results were obtained through a secondary data analysis from participants co-enrolled in the SYNERGIC and COMPASS-ND studies, across two time-points from the SYNERGIC study (baseline and post-training). All data processing and analyses were completed using Rstudio version 4.2.3 (RStudio Team, 2023). Descriptive statistics were carried out for each variable at each assessment timepoint. Outliers were identified using the boxplot method (i.e., values above the third quartile or below the first quartile range) and corrected using Winsorization (i.e., outliers were replaced with the next most extreme value). Each outcome measure was normally distributed (skew indices ranging from −0.20 to 0.47), though were mildly leptokurtic (kurtosis values ranged from 2.12–2.55). No transformations were computed since one of the primary goals of our analysis was to compare groups and assess changes over time. Transforming the data could lead to altered group distributions and relationships between variables, potentially distorting the natural structure of the data. By not transforming, we ensure that the comparisons across groups and time points remain valid and reflect the true underlying differences or trends present in the raw data. Group mean centering of continuous variables was conducted to improve accuracy of slope variance estimates and interpretation of main effects and interactions.

Chi square tests (for categorical variables) and one-way ANOVAs (for continuous variables) were conducted to determine if there were any differences between training groups at baseline. Bivariate correlations were conducted to determine if there were any associations between hearing measures and participants’ characteristics at baseline. Additionally, chi square tests and independent sample t-tests were conducted to determine if there were any significant differences in participant characteristics between males and females at baseline.

Separate linear mixed-effect models (LMM) were fitted for each of our outcome measures (i.e., gait speed, stride time, stride time variability, stride length, stride length variability, double-support time, cognitive accuracy) using the *lmer* (Bates et al., 2014) and *lmerTest* packages. The parsimonious model selection procedure (Bates et al., 2015) using a backwards elimination approach was utilized. The initial LMM included fixed effects for HHIE scores, CDTT SRTs, Task (single-task, dual-task), Condition (S1 Subtraction, S7 Subtraction, Semantic Fluency), Intervention (Multi-domain Training, Exercise, Placebo Training), Time (pre-, post-training), Age, Sex (male/female), Education, MoCA scores, and their interactions. Random intercepts, including the within-subject differences across testing sites, were included as a nested factor. Models were optimized based on LMM’s goodness of fit using the likelihood ratio test. Fixed effects and interactions that did not reliably contribute to model fit were removed, except for confounds (i.e., age, sex, education) to be consistent with previous literature, as well as instances where the fixed effects or interactions were necessary to evaluate our proposed research question (e.g., three-way interaction between hearing measures, intervention, and time). See Supplementary material for detailed model fit estimates.

Once the best fitting models were determined, *Estimates* and *p-values* were calculated from the *lmer* package (Bates et al., 2014) for continuous independent variables, and omnibus *F-* and *p*-values were calculated using the *Anova* function from the *car* package (Fox and Weisberg, 2019) for categorical independent variables. Kenward-Roger’s method was used to estimate degrees of freedom (Halekoh and Højsgaard, 2014). For the analyses involving continuous independent variables, relative effect sizes (i.e., proportion of variance explained by the given effect relative to the proportion of outcome variance unexplained) were computed as *f^2^* following Aiken et al. (1991) and West guidelines, where an effect size of 0.02 is considered small, 0.15 is considered medium, and 0.35 is considered large (Cohen, 1992). For our analyses involving categorical independent variables, effect sizes were computed as *η_p_^2^*, where a value of 0.01 indicates a small effect, 0.06 indicates a medium effect, and 0.14 indicates a large effect. Observation of statistically significant main effects or interactions were followed up by *post hoc* pairwise analyses with Bonferroni correction using the *emmeans* package (Lenth, 2023). Contrasts involving continuous variables were estimated using the *emtrends* function from the *emmeans* package.

## Results

### Baseline data inspection

Descriptive statistics and between-group analyses for the intervention arms and sex are shown for all background measures in Tables 1, 2, respectively. We did not find any significant differences across intervention arms or males and females for any of the background measures, with the exception of a significant difference in MoCA scores across training groups (Table 1). Post-hoc pairwise analyses showed that MoCA scores were marginally higher at baseline in the Multi-Domain group compared to the Placebo Training group, although this did not reach significance (*p* = 0.05). Nevertheless, MoCA performance was included as a covariate in all the linear mixed models.

**Table 2:** Descriptive statistics and independent sample *T*-tests/Chi-square tests for participant characteristics and hearing measures across males and females at baseline.

Variable	Males			Females			*t* or *χ^2^*	*p*	*d*
	*n*	*M*	*SD*	*n*	*M*	*SD*			
Age (years)	43	74.07	6.72	32	73.09	6.88	0.616	0.540	0.144
Education (years)	43	16.07	4.57	32	15.23	2.92	0.904	0.369	0.211
MoCA (/30)	43	22.63	2.82	32	23.44	3.16	−1.17	0.247	−0.270
HHIE-S (/40)	43	9.07	9.31	31	7.35	8.59	0.807	0.422	0.190
CDTT SRT (SNR)	30	−8.38	3.73	23	−9.48	3.89	1.04	0.302	0.289
Hearing Aid (*n*)	7			5			4.21	0.122	

MoCA, Montreal Cognitive Assessment; HHIE-S, Hearing Handicap Inventory for The Elderly – Screening Version; CDTT SRT, Canadian Digit Triplet Test – Speech Response Threshold; SNR, Signal to noise ratio.

In examining whether there were any associations between our hearing measures and participant characteristics at baseline, we found a significant positive correlation between CDTT SRTs and Age (*r* = 0.347, *p* < 0.05), and a significant negative correlation between CDTT SRTs and MoCA scores (*r* = −0.553, *p* < 0.001) such that hearing performance was worse (i.e., higher CDTT SRTs) in participants with lower MoCA scores and older age. There was also a significant positive correlation between HHIE-S scores and CDTT SRTs (*r* = 0.569, *p* < 0.001), such that a greater degree of self-reported hearing complaints (i.e., higher HHIE-S scores) was associated with poorer objective hearing performance (i.e., higher CDTT SRTs). Notably, the correlation between HHIE-S and CDTT SRTs differed across males (*r* = 0.720, *p* < 0.001) and females (*r* = 0.366, *p* = 0.094), such that greater hearing complaints (i.e., higher HHIE-S scores) were only associated with poorer hearing performance (i.e., higher CDTT SRTs) in male participants.

### Effect of hearing ability on single- and dual-task gait performance at baseline

In examining the effect of hearing ability on dual-task gait performance at baseline, we found significant effects of HHIE-S scores on temporal aspects of gait, gait stability, as well as gait DTCs (Table 3). Specifically, participants with greater hearing complaints (i.e., higher HHIE-S scores) had longer stride times, higher stride length variability, and higher DTCs for gait speed, stride time, and stride length variability. Similarly with the behavioral hearing data, we found significant effects of CDTT, such that participants with poorer objective hearing (i.e., higher CDTT SRTs) had slower gait speed, longer stride times, longer double-support time, and higher DTCs for gait speed, and double-support time (Table 3). We did not find any significant effects of hearing ability (measured with both self-report and behavioral testing) on single-task gait performance.

**Table 3:** Linear mixed models for all participants with hearing measures and MoCA included as fixed effects and dual-task gait performance as dependent variables.

Dependent variable	Fixed effects	*t (df)*	*β*	*SE*	*p*	*f^2^*
Gait speed (cm/s)	CDTT	−2.54 (45)	−40.98	16.12	0.010	0.09
	CDTT*MoCA	2.50 (45)	1.76	0.70	0.016	0.15
Gait speed DTC (%)	HHIE	2.56 (63)	3.37	1.37	0.017	0.05
	HHIE*MoCA	−2.44 (63)	−0.014	0.06	0.018	0.08
	CDTT	2.35 (45)	15.28	6.51	0.010	0.07
	CDTT*MoCA	−2.25 (45)	−0.64	0.28	0.030	0.08
Stride time (ms)	HHIE	2.29 (63)	43.20	18.84	0.025	0.07
	HHIE*MoCA	−2.39 (63)	−1.95	0.81	0.020	0.09
	CDTT	3.14 (45)	268.99	85.80	0.003	0.13
	CDTT*MoCA	−3.13 (45)	−11.75	3.75	0.003	0.15
Stride time DTC (%)	HHIE	2.72 (63)	2.51	0.92	0.009	0.06
	HHIE*MoCA	−2.76 (63)	−0.11	0.04	0.008	0.09
Stride length variability (CoV%)	HHIE	2.35 (63)	0.39	0.17	0.022	0.03
	HHIE*MoCA	−2.27 (63)	−0.02	0.01	0.026	0.07
	CDTT*MoCA	−2.22 (43)	−0.06	0.03	0.030	0.12
Stride length variability DTC (%)	HHIE	2.78 (65)	17.16	6.17	0.007	0.05
	HHIE*MoCA	−2.65 (63)	−0.70	0.27	0.010	0.07
Double-support time (ms)	CDTT	2.23 (43)	105.62	46.83	0.029	0.10
	CDTT*MoCA	−2.21 (43)	4.52	2.05	0.030	0.18
Double-support time DTC (%)	CDTT	2.21 (43)	21.27	9.64	0.033	0.07
	CDTT*MoCA	−2.04 (43)	−0.86	0.42	0.048	0.12

*f*^2^ = a measure of effect size indicating the proportion of variance explained by the given effect relative to the proportion of outcome variance unexplained; HHIE, Hearing Handicap Inventory for The Elderly – Screening Version; CDTT, Canadian Digit Triplet Test; MoCA, Montreal Cognitive Assessment. Other fixed effects included in the models were Age, Education, Sex, Dual-Task Condition (i.e., S1 and S7 subtractions, semantic fluency), and their interactions. Random effects included participant ID nested within the Training Site (i.e., Montreal, Western, University of British Columbia (UBC), Waterloo, Laurier). Degrees of freedom reflect the number of participants with available data for each measure. Some participants were missing CDTT data, resulting in smaller degrees of freedom compared to the HHIE-S.

### Effect of hearing ability on gait performance at baseline: interactions with cognitive status

Given the observed associations between hearing ability and dual-task gait, we then asked whether the severity of cognitive impairment would have an additional detrimental effect on dual-tasking. We found significant interaction effects of HHIE-S with MoCA scores for dual-task stride time and stride length variability, as well as DTCs for gait speed, stride time, and stride length variability (Table 3). Specifically, in participants with greater hearing complaints (i.e., higher HHIE-S scores), lower MoCA predicted poorer dual-task gait performance. Similarly, with the behavioral hearing data we found significant CDTT by MoCA interaction effects for dual-task gait speed, stride time, stride length variability, and double-support time, as well as DTCs for gait speed and double-support time (Table 3). Specifically, in participants with poorer hearing performance (i.e., higher CDTT SRTs), lower MoCA scores predicted poorer dual-task gait performance.

### Effect of hearing ability on gait performance at baseline: sex-specific interactions

Given the known sex differences in hearing loss, we also explored sex differences within our models and by stratifying by sex. We found significant main effects of HHIE-S scores, as well as HHIE-S and MoCA interactions for dual-task gait speed, stride time, and stride length variability, as well as the DTCs for gait speed, stride time, and stride length variability in males only (Table 4). Specifically, in male participants with greater hearing complaints (i.e., higher HHIE-S scores), dual-task gait performance was poorer, and there was a more negative effect of MoCA scores on gait performance (i.e., in males with more hearing complaints, lower cognitive status predicted poorer dual-task gait performance). Similarly, we found significant main effects of CDTT SRTs, as well as interaction effects between CDTT SRTs and MoCA scores on dual-task gait speed, stride time, and double-support time, as well as the DTCs for gait speed and double-support time in males only (Table 4). In males with poorer objective hearing performance (i.e., higher CDTT SRTs), gait performance was poorer, and there was a more negative effect of MoCA scores on gait performance. We did not find any significant effects of CDTT or HHIE-S on dual-task gait performance at baseline in females.

**Table 4:** Linear mixed models for males, with hearing measures and MoCA included as fixed effects and dual-task gait performance as dependent variables.

Dependent variable	Fixed effects	*t (df)*	*β*	*SE*	*p*	*f^2^*
Gait speed (cm/s)	HHIE	−2.36 (34)	−10.50	4.44	0.020	0.09
	HHIE*MoCA	2.33 (34)	0.45	0.19	0.026	0.15
	CDTT	−3.23 (34)	−60.71	18.83	0.004	0.19
	CDTT*MoCA	3.21 (34)	2.64	0.82	0.004	0.29
Gait speed DTC (%)	HHIE	3.33 (34)	5.80	1.74	0.002	0.14
	HHIE*MoCA	−3.22 (34)	−0.25	0.08	0.003	0.16
	CDTT	2.43 (24)	22.96	9.45	0.022	0.11
	CDTT*MoCA	−2.38 (24)	−0.98	0.41	0.030	0.13
Stride time (ms)	HHIE	2.68 (34)	60.83	22.71	0.011	0.12
	HHIE*MoCA	−2.69 (34)	−2.67	0.99	0.011	0.18
	CDTT	2.95 (24)	319.70	108.48	0.007	0.17
	CDTT*MoCA	−3.05 (24)	−14.47	4.74	0.005	0.22
Stride time DTC (%)	HHIE	3.67 (34)	4.15	1.13	<0.001	0.05
	HHIE*MoCA	−3.54 (34)	−0.17	0.05	0.001	0.08
Stride length variability (CoV%)	HHIE	3.04 (34)	0.66	0.22	0.004	0.12
	HHIE*MoCA	−2.78 (34)	−0.03	0.01	0.009	0.17
Stride length variability DTC (%)	HHIE	3.78 (34)	27.59	7.30	<0.001	0.15
	HHIE*MoCA	−3.45 (34)	−1.09	0.32	0.002	0.17
Double-support time (ms)	CDTT	2.26 (22)	141.47	62.74	0.030	0.14
	CDTT*MoCA	−2.30 (22)	−6.32	2.74	0.030	0.21
Double-support time DTC (%)	CDTT	2.42 (22)	34.77	14.32	0.025	0.10
	CDTT*MoCA	−2.32 (22)	−1.45	0.63	0.030	0.14

Note. f^2^ = a measure of effect size indicating the proportion of variance explained by the given effect relative to the proportion of outcome variance unexplained; HHIE = Hearing Handicap Inventory for The Elderly- Screening Version; CDTT = Canadian Digit Triplet Test; MoCA = Montreal Cognitive Assessment. Other fixed effects included in the models were Age, Education, Sex, Dual-Task Condition (i.e., S1 and S7 subtractions, semantic fluency), and their interactions. Random effects included participant ID nested within the Training Site (i.e., Montreal, Western, UBC, Waterloo, Laurier).

### Effect of hearing ability on single- and dual-task cognitive accuracy at baseline

In examining the effect of hearing ability on cognitive accuracy at baseline, we did not find any significant main effects of HHIE-S or CDTT, or any interactions with cognitive status. However, we found significant interaction effects between Sex and both behavioral and self-report hearing measures for single-task and dual-task performance. Specifically, in participants with fewer hearing complaints (i.e., lower HHIE-S scores), single-task cognitive accuracy was significantly lower in females (*M* = 0.41 corr/s, *SE* = 0.04), compared to males (*M* = 0.56 corr/s, *SE* = 0.04), *t*(64) = −3.02, *p* = 0.004, *η_p_^2^* = 0.10. Similarly, in participants with better objective hearing performance (i.e., lower CDTT SRTs), dual-task cognitive accuracy was significantly lower in females (*M* = 0.67 corr/s, *SE* = 0.05), compared to males (*M* = 0.82 corr/s, *SE* = 0.06), *t*(46) = −2.11, *p* = 0.04, *η_p_^2^* = 0.14. In contrast, in participants with a higher degree of perceived hearing loss (i.e., higher HHIE-S scores), dual-task cognitive accuracy was marginally lower in males (M = 0.74 corr/s, SE = 0.04) compared to females (*M* = 0.87 corr/s, *SE* = 0.05), *t*(65) = 1.95, *p* = 0.056, *η_p_^2^* = 0.07. Moreover, when the analyses were stratified by sex, there was a significant main effect of CDTT SRTs on dual-task cognitive accuracy for males, *t*(59) = −2.06, *p* = 0.04, *f^2^* = 0.02, such that in males, poorer objective hearing performance (i.e., higher CDTT SRTs) predicted lower accuracy scores (*β* = −0.07 corr/s, *SE* = 0.03). There was also a significant interaction effect of CDTT SRTs with Age on cognitive accuracy DTCs in males, *t*(28) = 3.12, *p* = 0.004, *f^2^* = 0.14, such that older age and a higher degree of hearing loss predicted higher cognitive DTCs (*β* = 1.76%, *SE* = 0.57).

### Effect of hearing ability on single- and dual-task performance from pre- to post-training

**Figure 2:**
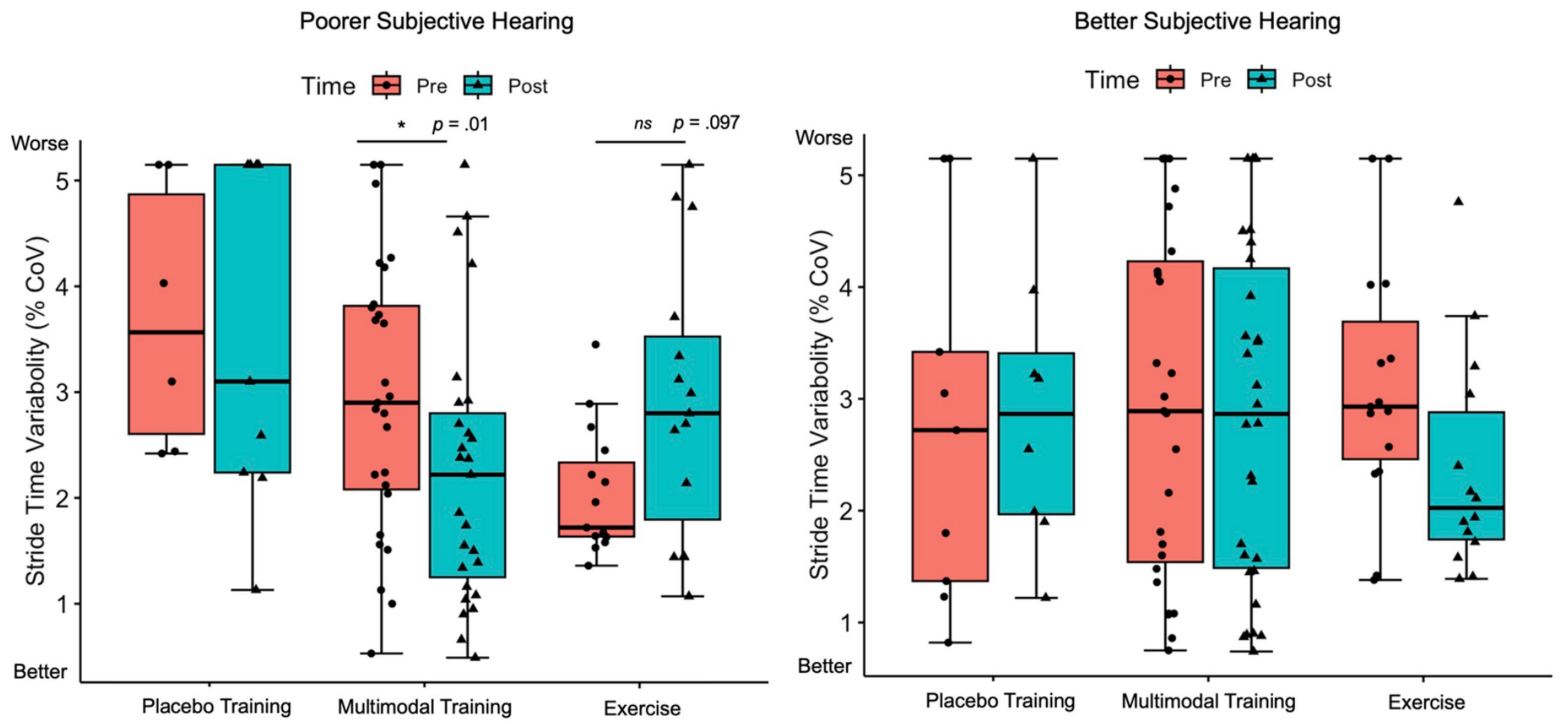
Linear mixed model of HHIE by time by training interaction effect on dual-stride time variability. Estimated marginal means of dual-task stride time variability (%CoV) following Placebo Training (balance and toning exercises + sham cognitive training), Multi-Domain Training (aerobic-resistance exercise + cognitive training) and Exercise (aerobic-resistance exercise + sham cognitive training) from pre- (orange) to post-training (blue) across participants with poorer self-reported hearing (left panel) and better self-reported hearing (right panel); The horizontal lines of the boxplots correspond to the 25th percentile, 50th percentile (median), and 75th percentile, respectively; The length of the box corresponds to the interquartile range (difference between 75th and 25th percentile); Each dot represents a data point for the serial 1 subtractions, serial 7 subtractions, and semantic fluency conditions for each participant; Error bars correspond to the 95% confidence intervals of the estimated marginal means; HHIE-S = Hearing Handicap Inventory for The Elderly – Screening Version; Poorer self-reported hearing = HHIE-S scores greater than 1SD above the mean; Better self-reported hearing = HHIE-S scores less than 1SD below the mean.

In examining whether hearing ability predicted single- and dual-task performance from pre- to post-training (Research Question 2), we found a significant HHIE-S by Time by Training Group interaction effect for dual-task stride time variability, *F*(2, 353) = 3.13, *p* = 0.045, *η_p_^2^* = 0.02. Specifically, in participants with a greater degree of hearing complaints (i.e., higher HHIE-S scores), there was a significant reduction (i.e., improvement) in dual-task stride time variability only following Multi-Domain Training, *Δ* = −0.54% CoV, *SE* = 0.21, *p* = 0.01 (Figure 2). We did not find any significant interactions between hearing ability and change in single-task gait, single- and dual-task cognitive accuracy, or DTCs following training. Supplementary analyses examining hearing loss categorically using an abbreviated pure-tone audiometry assessment revealed similar results, such that dual-task stride time, stride time variability, and double-support time significantly improved in participants with hearing loss, but not in participants with normal hearing, regardless of training modality (i.e., Placebo Control, Multi-Domain, Exercise Training). Additionally, following Multi-Domain Training, dual-task stride length significantly improved in participants with hearing loss, and marginally improved in participants with normal hearing (see Supplementary material).

### Effect of hearing ability on dual-task gait performance from pre- to post-training: interactions with cognitive status

**Figure 3:**
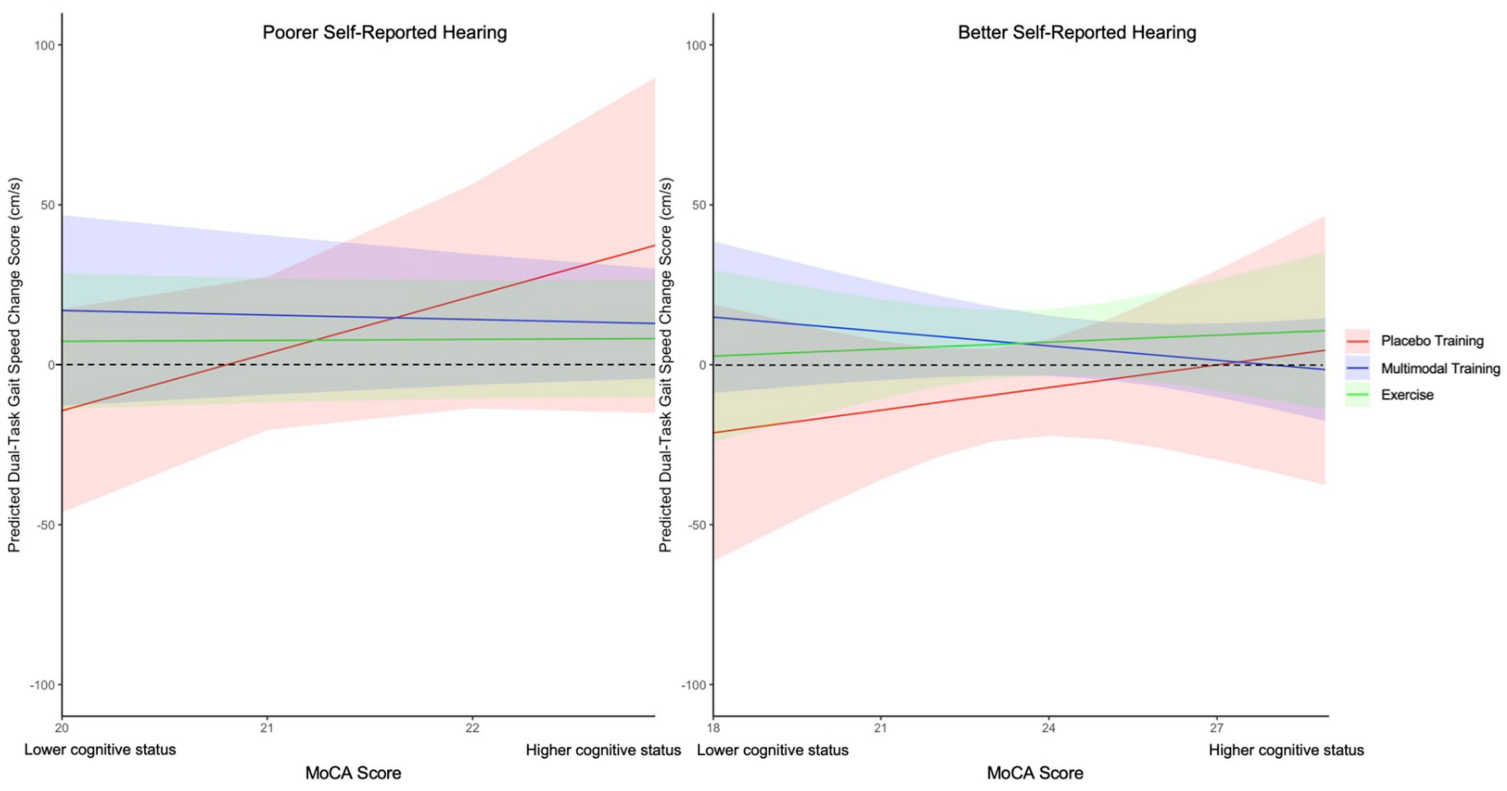
Linear mixed model of HHIE by training by MoCA interaction effect for dual-task gait speed change scores from pre- to post-training. MoCA *β* estimates of dual-task gait speed change scores (ms) following Placebo Training (red), Multi-Domain Training (blue), and Exercise (green) across Poorer Self-Reported Hearing (left) and Better Self-Reported Hearing (right); Placebo Training consisted of balance and toning exercises with sham cognitive training; Multi-Domain Training consisted of aerobic-resistance exercise with cognitive training, Exercise consisted of aerobic-resistance exercise with sham cognitive training; Error bars correspond to the 95% confidence intervals of the estimates; MoCA = Montreal Cognitive Assessment; HHIE = Hearing Handicap Inventory for The Elderly – Screening Version; Better Self-Reported Hearing = HHIE-S scores less than 1SD below the mean; Poorer Self-Reported hearing = HHIE-S scores greater than 1SD above the mean; Dotted line represents point of inflection for improvement or worsening of dual-task gait speed following training (i.e., scores above zero indicate improvement and scores below zero indicate worsening). Dual-task performance was aggregated across the Serial 1 subtractions, Serial 7 subtractions, and Semantic fluency conditions.

Given the baseline findings demonstrating interactions between MoCA scores and our hearing measures on dual-task gait performance, we examined whether individuals with a greater severity of hearing loss and poorer cognitive functioning benefited differentially from the training compared to those with less severe impairments. We found significant HHIE-S by MoCA by Training interaction effects for dual-task gait speed, *F*(2, 54) = 4.10, *p* = 0.021, *η_p_^2^* = 0.13 (Figure 3), stride time, *F*(2, 53) = 4.98, *p* = 0.01, *η_p_^2^* = 0.16, and double-support time, *F*(2, 51) = 5.72, *p* = 0.006, *η_p_^2^* = 0.18. Specifically, in participants with greater hearing complaints (i.e., higher HHIE-S scores), lower MoCA scores significantly predicted worsened dual-task gait speed (*β* = 13.54 cm/s, *SE* = 5.47, *p* = 0.017), stride time (*β* = −70 ms, *SE* = 26.68, *p* = 0.01) and double-support time (*β* = −69.84 ms, *SE* = 26.21, *p* = 0.01) following Placebo Training.

**Figure 4:**
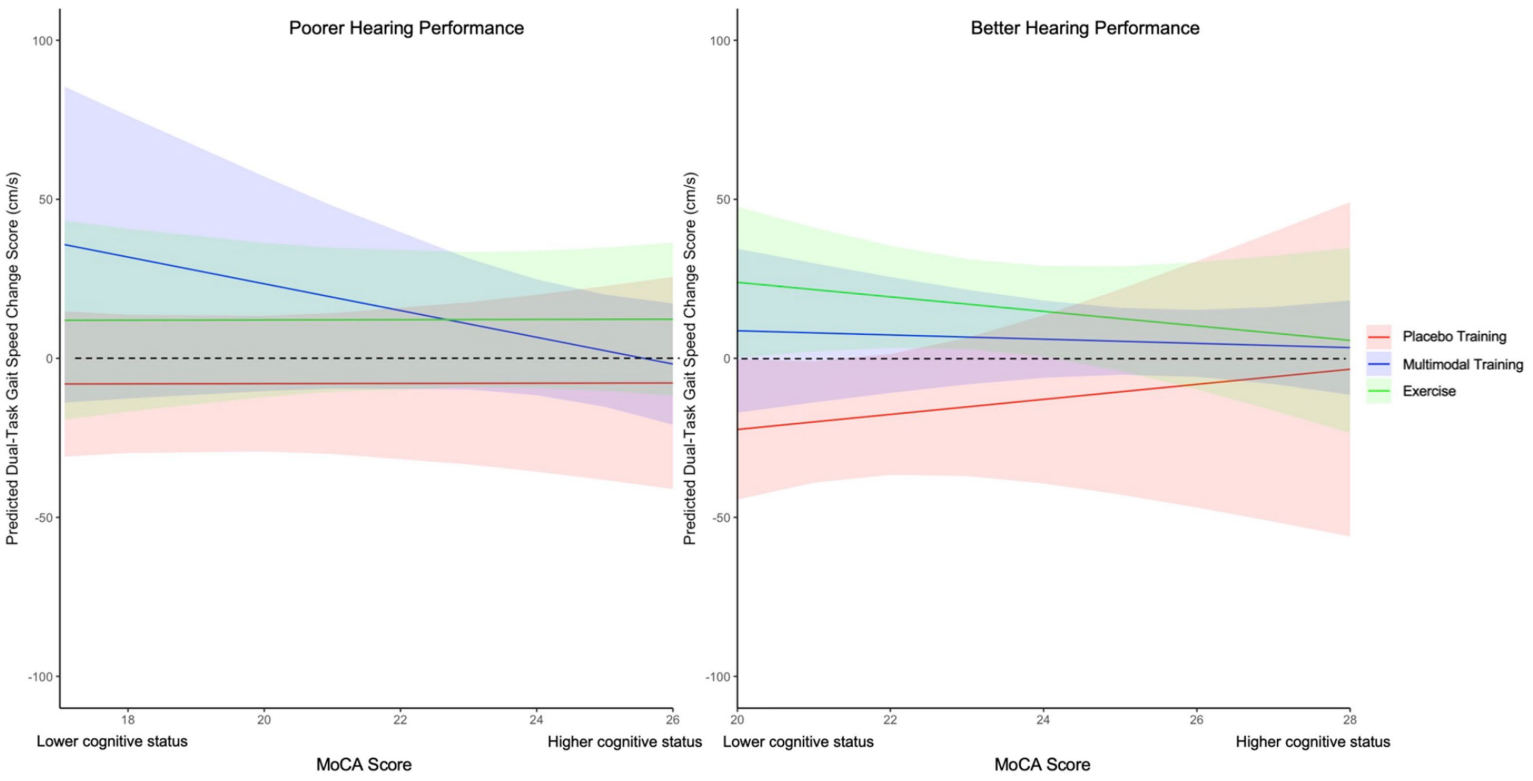
Linear mixed model of CDTT by training by MoCA interaction effect for dual-task gait speed change scores from pre- to post-training. MoCA *β* estimates of dual-task gait speed change scores (ms) following Placebo Training (red), Multi-Domain Training (blue), and Exercise (green) across Poorer Hearing Performance (left) and Better Hearing Performance (right); Placebo Training consisted of balance and toning exercises with sham cognitive training; Multi-Domain Training consisted of aerobic-resistance exercise with cognitive training; Exercise consisted of aerobic-resistance with sham cognitive training; Error bars correspond to the 95% confidence intervals of the estimates; MOCA = Montreal Cognitive Assessment; CDTT SRT = Canadian Digit Triplet Test Speech Response Threshold; Better Hearing Performance = SRTs less than 1SD below the mean; Poorer Hearing Performance = SRTs greater than 1SD above the mean; Dotted line represents point of inflection for improvement if worsening of dual-task gait speed following training (i.e., scores above zero indicate improvement and scores below zero indicate worsening). Dual-task performance was aggregated across the Serial 1 subtractions, Serial 7 subtractions, and Semantic fluency conditions.

Additionally, we found significant CDTT by MoCA by Training interaction effects for dual-task gait speed, *F*(2, 37) = 3.73, *p* = 0.033, *η_p_^2^* = 0.17 (Figure 4), stride time, *F*(2, 34) = 3.98, *p* = 0.028, *η_p_^2^* = 0.19, and double-support time, *F*(2, 36) = 5.92, *p* = 0.006, *η_p_^2^* = 0.25. Specifically, in participants with poorer CDTT performance (i.e., higher CDTT SRTs), lower MoCA scores significantly predicted improved dual-task gait speed (*β* = −4.08 cm/s, *SE* = 1.37, *p* = 0.005), stride time (*β* = 32.45 ms, *SE* = 12.85, *p* = 0.016) and double-support time (*β* = 24.59 ms, *SE* = 7.40, *p* = 0.002) following Multi-Domain Training. In contrast, in participants with poorer CDTT performance (i.e., higher CDTT SRTs), lower MoCA scores predicted worsened dual-task gait speed (*β* = 23.23 cm/s, *SE* = 7.72, *p* = 0.005), stride time (*β* = −198.65 ms, *SE* = 70.5, *p* = 0.008) and double-support time (*β* = −110.05 ms, *SE* = 42.39, *p* = 0.014) following Placebo training.

### Effect of hearing ability on dual-task gait performance from pre- to post-training: sex-specific interactions

To examine whether the effect of hearing ability on dual-task outcomes following training differed across males and females, we stratified our analyses by sex. We found a significant HHIE-S by Time interaction effect in female participants for stride time, *F*(1, 140) = 5.39, *p* = 0.021, *η_p_^2^* = 0.04, such that females with greater self-reported hearing complaints (i.e., higher HHIE-S scores), showed a significant reduction (i.e., improvement) in stride time, regardless of intervention arm (*Δ* = −42.45 ms, *SE* = 13.0, *p* = 0.001). In contrast to this, analyses involving behavioral hearing performance in females revealed significant CDTT by Time by Training interaction effects in the opposite direction for double-support time, *F*(2, 101) = 8.48, *p* < 0.001, *η_p_^2^* = 0.14, and stride time, *F*(2, 101) = 6.12, *p* = 0.003, *η_p_^2^* = 0.11. Specifically, in female participants with better objective hearing performance (i.e., lower CDTT SRTs), stride time (Δ = −79.2 ms, *SE* = 21.1, *p* < 0.001) and double-support time (Δ = −46.18 ms, *SE* = 10.96, *p* < 0.001) significantly improved following Multi-Domain Training, though significantly worsened following Placebo Training (stride time: Δ = 116.8 ms, *SE* = 31.5, *p* < 0.001; double-support time: Δ = 53.28 ms, *SE* = 16.31, *p* = 0.002). We additionally found a significant CDTT by Time interaction effect in females for dual-task cognitive accuracy, *F*(1, 107) = 8.21, *p* = 0.005, *η_p_^2^* = 0.07, such that accuracy scores significantly improved following training in participants with better objective hearing performance (i.e., lower CDTT SRTs), regardless of training modality (Δ = 0.19 corr/s, *SE* = 0.09, *p* = 0.036).

In male participants, we did not find any significant effects of self-reported hearing loss on the degree of improvement in dual-task performance following training; however, we found significant effects for our behavioral hearing measure. Specifically, we found significant CDTT by Time by Training Group interaction effects for dual-task gait speed, *F*(2, 142) = 3.91, *p* = 0.022, *η_p_^2^* = 0.05, and stride length, *F*(2, 140) = 7.77, *p* < 0.001, *η_p_^2^* = 0.10, such that males with poorer objective hearing performance (i.e., higher CDTT SRTs) only showed significant improvements in gait speed (Δ = 9.51 cm/s, *SE* = 4.10, *p* = 0.022), and stride length (Δ = 8.09 cm, *SE* = 2.54, *p* = 0.002) following Multi-Domain Training.

## Discussion

By examining a subset of participants with MCI from the SYNERGIC trial who were co-enrolled in a longitudinal study that included a hearing evaluation, we examined whether hearing ability (including participants with clinical and subclinical hearing loss) affected dual-task performance at baseline and following 20-weeks of physical exercise training, alone or in combination with computerized cognitive training. Given the known sex differences in hearing loss and dementia, we further sought to examine whether any sex-specific interactions emerged with hearing ability on the degree of dual-task improvements following training.

**Figure 5:**
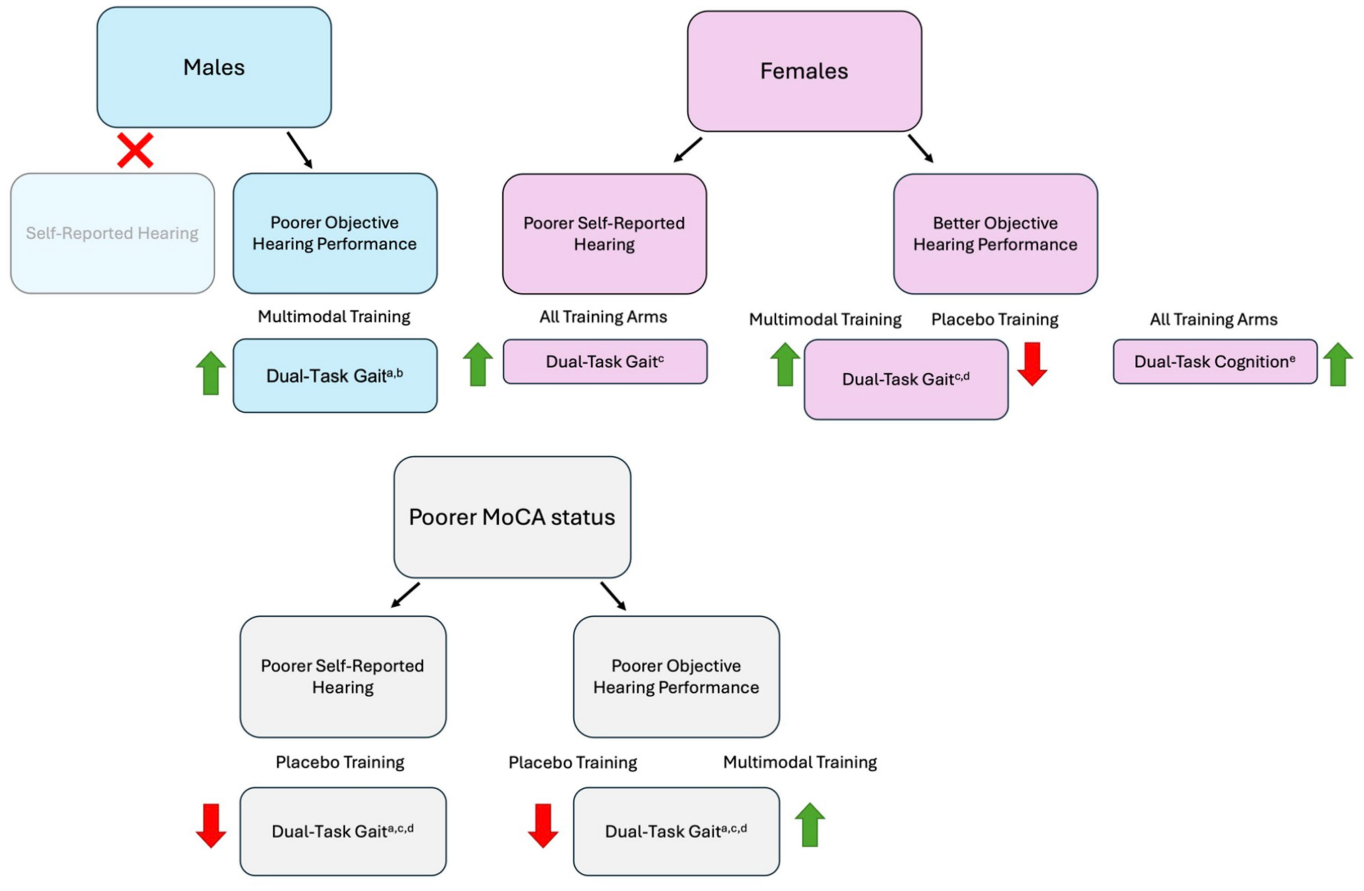
Schematic overview of training results according to sex and cognitive status interactions. The green upward arrows represent improved dual-task performance following training, whereas the red downward arrows represent a reduction in dual-task performance following training; ^a^gait speed, ^b^stride length, ^c^stride time, ^d^double-support time, ^e^cognitive accuracy; Placebo Training consisted of balance and toning exercises with sham cognitive training; Multi-Domain Training consisted of aerobic-resistance exercise with cognitive training: MOCA = Montreal Cognitive Assessment; Objective hearing performance was measured using the Canadian Digit Triplet Test; Self-reported hearing was measured using the Hearing Handicap Inventory for The Elderly – Screening Version.

Our baseline results demonstrated that MCI participants with a greater degree of hearing loss (measured by both self-report and behavioral testing) had poorer dual-task gait performance, including temporal aspects of gait (e.g., gait speed, stride time) as well as gait stability (e.g., stride length variability, double-support time). The relationship between hearing loss and dual-task performance at baseline appeared to be largely driven by the male participants. Regarding our training results, we found that dual-task gait significantly improved following multi-domain exercise and cognitive training in participants with a greater degree of self-reported hearing complaints, and in participants with poorer cognitive status combined with poorer objective hearing performance. We further found that training efficacy was moderated by interactions between sex and hearing ability, such that dual-task gait improved in males with poorer objective hearing and in females with better objective hearing following multi-domain training, as well as in females with greater self-reported hearing complaints (regardless of training arm). See Figure 5 for a schematic overview of the training results.

### Effect of hearing ability on dual-task performance at baseline and following training

As anticipated, we found a greater magnitude of improvement in dual-task gait stability (i.e., stride time variability) following combined physical exercise and cognitive training in participants with poorer self-reported hearing. Other research has shown similar synergistic effects on cognition and mobility in cognitively unimpaired older adults (Bherer et al., 2021) and older adults with MCI (Castellote-Caballero et al., 2024). Our results are novel in showing a synergistic effect of training on cognitive-motor dual-task outcomes in MCI participants with a greater degree of self-reported hearing loss. These findings are partially consistent with our previous training work in cognitively unimpaired older adults (Bruce et al., 2019b). Specifically, cognitively unimpaired older adults with hearing loss had similar levels of dual-task improvement following either a simultaneously or sequentially delivered physical exercise and cognitive training program, whereas older adults with normal hearing only improved following the sequential training format (Bruce et al., 2019b). Participants with hearing loss may have had greater opportunity for improvement due to greater dual-task costs observed at baseline under challenging listening conditions compared to normal hearing participants (Bruce et al., 2019a), allowing either approach to be beneficial.

Several mechanistic theories have been proposed to explain the association between hearing loss and reduced cognitive functioning (e.g., information degradation, sensory deprivation; Schneider and Pichora-Fuller, 2000), which may inform our training-related findings. For example, the sensory deprivation hypothesis suggests that over time, auditory deprivation may lead to temporal lobe atrophy and the re-allocation of attentional resources to compensate for an impoverished signal (Lin et al., 2014; Peelle and Wingfield, 2016; Powell et al., 2022). Dual-task interference may therefore be exacerbated in individuals with a higher degree of hearing loss due to alterations in brain structure that lead to changes in higher level brain function. Specifically, hearing loss may cause increased competition for cognitive control regions while dual-tasking due to the re-allocation of attentional resources that occurs over time to support auditory processing (Powell et al., 2022). In the current study, we found that males with a higher degree of hearing loss (measured by both self-report and behavioral testing) had poorer dual-task gait and cognitive performance at baseline. These findings are consistent with a growing body of literature, which suggests that older adults with hearing loss have reduced dual-task gait and balance, as well as poorer working memory performance, compared to individuals with normal hearing (Wunderlich et al., 2024).

Given our baseline findings demonstrating poorer dual-task performance in participants with poorer hearing, our training results appear largely consistent with a compensation account of training (Lövdén et al., 2012). This theory posits that when intervention task demands exceed available resources, repeated training induces neural plasticity and promotes a higher level of change in performance following training. Our results are also consistent with compensatory scaffolding theories (Li et al., 2018; Reuter-Lorenz and Park, 2014), such that Multi-Domain Training may have increased the capacity for neural scaffolding in MCI participants with a greater degree of hearing loss, due to potential alterations in brain structure and function caused by changes in auditory processing over time.

### Effect of hearing ability on dual-task performance following training: sex-specific interactions

Given potential sex differences in hearing loss and MCI, we further examined whether any sex-specific interactions emerged with hearing ability on the degree of dual-task improvements following training. When we stratified our analyses by biological sex, differential effects emerged depending on the hearing measure used and the sex of the participants. Specifically, we found a synergistic effect of Multi-Domain Training on dual-task performance in male participants with poorer objective hearing performance, as dual-task gait speed and stride length only significantly improved following physical exercise combined with cognitive training. While research has shown that hearing loss is more prevalent and more severe in males compared to females (Nolan, 2020), in the current study, we did not find any significant differences in hearing ability between sexes. Additionally, no other participant characteristics significantly differed between sexes, including age, years of education, global cognitive status, or hearing aid use. As such, it remains unclear what is driving these sex effects on dual-task performance before and after training.

One possible interpretation is that given that hearing loss tends to have an earlier age of onset in males compared to females, there may have been more time for structural and functional brain changes to occur, leading to increased competition for cognitive control regions while dual-tasking. This is consistent with our baseline findings, which revealed that the association between hearing loss severity and dual-task performance was largely driven by the male participants. This idea echoes previous proposals of gradual age-related sensory and cognitive declines occurring in midlife followed by functional adaptations in the form of changes to attentional allocation and neural reorganization (Li and Lindenberger, 2002). Additionally, in a more recent longitudinal study by Boons et al. (2024), poorer peripheral hearing acuity was found to be associated with faster microstructural deterioration over time in a white matter tract involved in auditory processing. The link between hearing acuity and white matter integrity was stronger in low-frequency hearing thresholds, which tend to deteriorate at a faster rate in males compared to females (Dillard et al., 2024). Furthermore, using a similar sample as the current study, we have previously found sex differences in brain activation in relation to physical frailty (Bray et al., 2023b). Specifically, in male MCI participants, stronger functional brain connectivity between the right hippocampus and temporal gyrus positively correlated with increased frailty. While this previous study did not examine hearing loss specifically, it is possible that the male participants in our sample underwent greater structural and functional brain changes due to alterations in hearing capacity compared to females, leading to greater dual-task deficits at baseline and a greater opportunity for improvement following Multi-Domain Training. However, further research is needed to support this interpretation. Finally, gender-related factors such as occupation (e.g., fields with increased noise exposure) and lifestyle choices (e.g., smoking) may have differed across men and women in our sample and may have interacted with hearing capacity (Reavis et al., 2023). Nonetheless, *post hoc* analyses examining sex- and gender-based differences across other risk factors for dementia (e.g., hypertension, depression, smoking, diabetes) did not reveal any significant differences. Further research may therefore be warranted to better understand whether the sex-related interactions observed in the current study are due to biological sex differences or gender-related lifestyle factors.

In contrast to our findings in males, in female participants with better objective hearing performance, there was a synergistic effect of Multi-Domain Training on dual-task gait, and a positive impact of training on dual-task cognition, regardless of the training domain. However, female participants with greater subjective hearing complaints showed significant improvements in dual-task gait following training, regardless of the intervention arm. One potential reason for the disparity in our results across the self-reported and behavioral hearing measures may be that there was a weak association between hearing measures in our female participants. Indeed, we only found a significant correlation between HHIE-S and CDTT SRTs in male participants, such that a greater degree of self-reported hearing loss was associated with poorer CDTT performance. Further analyses categorizing hearing loss according to performance across each hearing measure (i.e., hearing loss was categorized as HHIE-S > 10; Ventry and Weinstein, 1983; CDTT SRTs > − 10 dB SNR; Al-Yawer et al., 2023) demonstrated a significant difference across measures in females (*χ^2^* = 5.62, *p* = 0.018), but not in males (*χ^2^* = 0.50, *p* = 0.481). As such, it is possible that female participants endorsed an elevated level of hearing complaints amidst relatively preserved hearing capacity. Interestingly, other research in cognitively unimpaired older adults has found that males report greater perceived hearing difficulties compared to females, even after controlling for hearing loss and hearing thresholds (Hämäläinen et al., 2021; Humes, 2021). Our findings also echo what has been reported in the frailty literature. Specifically, while there is evidence of a male–female health survival paradox, such that females become frail earlier yet live longer than males (Gordon et al., 2017), research now suggests that this might be due in part to symptom over-reporting or poorer perception of self-rated health in females (Park and Ko, 2021).

Another interpretation of our results can be made by considering the baseline results, which demonstrated that in participants with better objective hearing performance, females had poorer dual-task cognitive accuracy compared to males. Consistent with the compensation account of training (Lövdén et al., 2012), female participants may have had more opportunity for improvement following active training; however, they may have been at a greater disadvantage following the placebo training, due to the typical trajectory of cognitive, physical, and neural decline in MCI over time. This is consistent with a recent study which showed that in older adults with amnestic MCI, white matter fiber density declined at a faster rate following 6 months of sham cognitive training (i.e., completing crossword puzzles) compared to active cognitive training (i.e., memory and executive function training; Gozdas et al., 2024).

### Effect of hearing ability on dual-task performance following training: interactions with cognitive status

Given the observed associations between hearing ability and dual-task performance, we wondered whether the severity of cognitive impairment would have an additional detrimental effect on dual-tasking, and additionally, if the severity of cognitive impairment influenced training-related outcomes. Our results demonstrated that at baseline, in participants with poorer hearing, lower cognitive status (i.e., lower MoCA scores) predicted poorer dual-task gait performance (i.e., gait speed, stride time, stride length variability, double-support time, gait DTCs). We further found a synergistic effect of Multi-Domain Training in participants with poorer cognitive status combined with poorer objective hearing on temporal aspects of gait, as well as gait stability. Specifically, in participants with poorer objective hearing performance, poorer cognitive status predicted improvements in dual-task gait speed, stride time, and double-support time, only following physical exercise combined with cognitive training. Additionally, we found that following the Placebo Training, dual-task gait performance (i.e., gait speed, stride time, double-support time) declined in participants with poorer hearing and *lower* cognitive status, though improved in participants with poorer hearing and *higher* cognitive status.

These results are novel in that they provide insight into the types of training that promote the greatest magnitude of improvements depending on individual characteristics by considering hearing ability and cognitive functioning. According to the capacity theory, dual-tasking leads to competition for limited resources, causing decrements in performance in one or both tasks (Salthouse et al., 1984; Woollacott and Shumway-Cook, 2002; Wright, 1981). As attentional resources may need to be re-allocated due to structural brain changes caused by alterations in auditory processing over time, dual-task effects are likely to be exacerbated in individuals with a greater degree of hearing loss. In addition to hearing loss, participants with lower cognitive status might have fewer cognitive resources to draw from while dual tasking, leading to an added detriment to dual-task performance. Indeed, research has shown that poor cognition mediates the relationship between sensory functioning and gait speed in older adults (Huang et al., 2019). This is consistent with our baseline findings, which demonstrated that participants with reduced cognitive and hearing functioning had poorer dual-task gait performance. These participants may have therefore had greater opportunity for improvement following physical exercise combined with cognitive training, consistent with a compensation account of training (Lövdén et al., 2012). These results are also consistent with our previous training work, where we demonstrated that in cognitively unimpaired older adults, participants with lower MoCA scores at baseline had a greater magnitude of improvement in dual-task working memory performance following training (Downey et al., 2022). Additionally, in the current study, participants with poor hearing and lower cognitive status appeared to be most disadvantaged following the Placebo Training. These results may suggest that participating in balance, stretching, and toning exercises, in addition to completing basic computer tasks does not prevent or slow the progression of cognitive and physical decline in this particularly at-risk group (i.e., low cognitive status, poor hearing).

We further found that in participants with poor hearing, higher cognitive status predicted a greater degree of dual-task gait improvements following the Placebo Training. Other research has shown that gross motor training, involving balance and stretching exercises, improves executive functioning (Berryman et al., 2014) and dual-task working memory performance (Downey et al., 2022) in cognitively unimpaired older adults. As such, it is plausible that a certain level of cognitive functioning is required to elicit benefits from the types of activities included in our Placebo Training. Additionally, research suggests that individuals with hearing loss may withdraw from social interactions due to increased listening effort, which makes conversing fatiguing (Pichora-Fuller et al., 2016). This may result in isolation and reduced physical activity, further contributing to cognitive and motor limitations due to hearing loss. For example, Mikkola et al. (2016) demonstrated that in cognitively unimpaired older adults, hearing loss was associated with reduced participation in group activities outside of the home. As all the interventions in the current study were in-person and comprised of small groups, those with hearing loss in the placebo treatment may have experienced an increase in their physical activity levels and number of social interactions, which may have contributed to improvements in dual-task gait. Notably, this may have only been observed in participants with poor hearing and *higher* cognitive status as these individuals might have been able to draw on additional cognitive resources to support dual-task gait performance. Future research into the effect of cognitive and physical training on dual-task outcomes using an at-home intervention protocol may be warranted, to better control for these factors (e.g., Downey et al., 2023).

### Limitations

The results of this study should be considered in light of some limitations. While our overall sample size was satisfactory, our intervention groups were unequal in size, with fewer participants in the Placebo Training group. As such, careful consideration must be given when interpreting the results from the Placebo Training group, particularly when the training analyses were stratified by sex as there were fewer female participants compared to males. To preserve statistical power, we were also unable to disaggregate our analyses according to MCI subtype, which may further limit some of the interpretations and implications of our results (e.g., comparing amnestic vs. non-amnestic MCI). Additionally, our sample was comprised of normal hearing older adults, as well as individuals with clinical and subclinical hearing loss. It is important to note that poorer hearing scores do not necessarily indicate clinical hearing loss; for example, participants may have scored higher on the HHIE-S or had higher CDTT SRTs relative to our overall sample, though some participants might not have reached the clinical cut-off to be considered hearing impaired. Nevertheless, evidence suggests that subclinical hearing loss can negatively impact cognitive functioning, consistent with our results (Golub et al., 2020). Supplementary analyses categorizing hearing loss according to an abbreviated pure-tone audiometry assessment revealed similar results as the current study (see Supplementary material), further bolstering our interpretations made using the continuous hearing measures. Other factors that may have influenced our results include the high variability in HHIE-S scores, reflecting the wide range of hearing ability in the sample, which may have increased sensitivity to detect associations but also added uncertainty to subgroup analyses. In addition, for a small subset of participants, longer intervals between hearing and dual-task assessments may have reduced the specificity of hearing status at the time of gait testing and attenuated observed associations, although these intervals did not differ by sex or intervention group.

Furthermore, around 16 % of our sample wore hearing aids. As there is some evidence to suggest that improved signal quality (e.g., by hearing aid use) may benefit cognitive performance (e.g., Lin et al., 2023; Sanders et al., 2021), and cognitive dual-task performance (Soylemez et al., 2024) in older adults with hearing loss, our results may represent a more conservative estimate of participants’ dual-task performance before and after training. Additionally, the behavioral hearing assessments were conducted without hearing aids, while the dual-task experiment was completed with hearing aids, which may have potentially interfered with the relationships observed. We note however that there was no record of the type of hearing aids worn or how long the hearing aids were used by the participants, so more detailed research examining the effect of hearing aid use on dual-task performance may be warranted.

Another potential limitation of the study design was that there was no active cognitive training paired with the balance and toning exercises. Due to this, it remains unclear whether the benefits observed in dual-task performance were specific to the multi-domain nature of the training, or whether improvements were driven by the cognitive training component. Using a balanced training design, where participants are allocated to four different combinations of interventions (i.e., active cognitive training vs. sham cognitive training; aerobic-resistance exercise vs. stretching and toning exercises), researchers have previously found mixed effects in cognitively unimpaired older adults. Specifically, Fraser et al. (2017) demonstrated a similar magnitude of improvement in cognitive-motor dual-task performance following any combination of active and control interventions, whereas Bherer et al. (2021) only found improvements in cognitive dual-task performance following cognitive training, or multi-domain cognitive training with physical exercise. Notably, there is significant heterogeneity across different multi-domain interventions, highlighting the potential need for individually tailored interventions to optimize training gains (Barha et al., 2022; Izquierdo et al., 2025). Further research with MCI individuals is needed to elucidate the effect of hearing ability on cognitive training efficacy more specifically.

Additionally, while we analyzed cognition and its interactions with hearing ability, we did not examine broader motor functioning in the present study. While additional measures such as the 6-Minute Walk Test and Short Physical Performance Battery (SPPB) were collected as part of the SYNERGIC trial, this data was not included in our analyses to preserve parsimony in the linear mixed-effects models and maintain focus on dual-task performance. We recognize that changes in motor status are an important factor influencing cognitive-motor interactions, particularly in older adults with MCI and hearing loss, and future work should consider how motor function interacts with sensory and cognitive factors to affect training outcomes.

Finally, our dual-task design was comprised of three different secondary cognitive tasks (i.e., serial 1 subtractions, serial 7 subtractions, semantic fluency), although our results were aggregated across these measures. Research suggests that low cognitive loads may improve gait and balance performance compared to single-task conditions in cognitively unimpaired younger and older adults, whereas motor dual-task costs increase at higher levels of cognitive task complexity (Huxhold et al., 2006; Li et al., 2012; Lövdén et al., 2008). While aggregating across different levels of secondary cognitive task complexity may have impacted our results to some degree, Condition was included as a factor in our linear mixed effect models, and was removed from the models if it did not significantly contribute to model fit, or did not interact with our other key factors (e.g., hearing ability, sex). Additionally, a study by Hunter et al. (2018) demonstrated that low levels of cognitive task complexity (i.e., Serial 1 subtractions) negatively impacted gait velocity compared to single-task gait in MCI participants, but not in cognitively unimpaired older adults. As such, the point of inflection, wherein the cognitive task begins to interfere with mobility performance, appears to occur at lower levels of cognitive task complexity in cognitively impaired older adults, which bolsters our decision of aggregating the results across the three secondary cognitive tasks.

## Conclusion

The current study provides novel insights into the types of training that promote the greatest magnitude of improvements in cognitive-motor dual-task performance in older adults with MCI, by considering interactions between hearing ability, biological sex, and cognitive functioning. Overall, our results suggest that there is a potentially synergistic effect of physical exercise combined with cognitive training in older adults with MCI who have a greater degree of hearing loss. This effect appears to be strongest in males, as well as in individuals with poorer hearing combined with lower cognitive status. Our training results in females varied according to the hearing measure used, which may be explained by the poorer concordance across the self-report and behavioral hearing measures in females than in males, with some females endorsing an elevated level of self-reported hearing difficulty despite relatively preserved performance on the CDDT hearing test. These findings further highlight the clinical relevance of using both self-reported and objective hearing measures to identify older adults with MCI who are most likely to benefit from targeted cognitive-motor interventions.

The detriment to dual-task performance may be exacerbated in MCI individuals with the additional challenge of hearing loss due to associated neural alterations and cortical reorganization. This challenge appeared to be reduced by Multi-Domain physical exercise and cognitive training, whereby increasing physical and higher-level cognitive resources may have enhanced compensatory scaffolding. Older adults with MCI are at a greater risk for falls compared to cognitively healthy older adults and, conversely, slow gait speed predicts cognitive decline (Montero-Odasso et al., 2012b). As such, preserving dual-task gait and cognitive performance through physical exercise in combination with cognitive training may be critical for mitigating further cognitive and physical decline during this early prodromal stage, particularly in individuals with hearing loss.

## Data Availability

The raw data supporting the conclusions of this article will be made available by the authors, without undue reservation.
