# Supplementary material for: The effect of hearing ability on dual-task performance following multi-domain training in older adults with mild cognitive impairment: findings from the SYNERGIC trial
Source: Front Aging Neurosci. 2026 Jan 30;17:1716733. doi: 10.3389/fnagi.2025.1716733 (PMC12901436; doi:10.3389/fnagi.2025.1716733)
Supplement: Supplementary file 1 [file Data_Sheet_1.pdf]

Supplementary Material

Materials

Background Measures

An abbreviated pure-tone audiometry assessment was conducted as a screening procedure in the COMPASS-ND study using a GSI 18 audiometer in a quiet clinical examination room. Participants were assigned to one of two hearing loss categories based on their ability to detect 2-kHz pure tones. Participants who were able to detect a 2-kHz tone at 25 dB HL on at least one trial in either one or both ears were assigned to category 1 ("Normal Hearing"), whereas participants who were unable to detect a 2-kHz tone at 25 dB HL in both ears were assigned to category 2 ("Hearing Impaired"). This methodology has been validated in a previous study examining the effect of hearing loss on cognition in MCI participants using data from the COMPASS-ND study (Al-Yawyer et al., 2022).

Statistical Analyses

The factors included in each linear mixed model, as well as the model fit estimates, for each outcome measure at baseline and following training are described below. Note that conditional R^2 indicates the amount of variance explained by both fixed and random factors, while marginal R^2 indicates the amount of variance explained by only the fixed factors.

Baseline Models

Cognitive accuracy. The selected mixed effect model (conditional R^2 = 0.73, marginal R^2 = 0.66) to examine whether hearing loss severity (as measured with HHIE) significantly predicted cognitive accuracy at baseline (Hypothesis 1) included the following fixed effects: HHIE x Condition, HHIE x Sex, HHIE x Education, Condition x Education, and MoCA x Age, and the following random intercepts: Subject nested within Testing Site. The selected mixed effect model

(conditional R^2 = 0.72, marginal R^2 = 0.64) to examine whether hearing loss severity (as measured with CDTT) significantly predicted gait speed at baseline (Hypothesis 1) included the following fixed effects: CDTT x Condition, CDTT x Sex, Age, MoCA, and Education, and the following random intercepts: Subject nested within Testing Site.

Gait speed. The selected mixed effect model (conditional R^2 = 0.89, marginal R^2 = 0.17) to examine whether hearing loss severity (as measured with HHIE) significantly predicted gait speed at baseline (Hypothesis 1) included the following fixed effects: HHIE x Condition, HHIE x MoCA, Age, Education, and Sex, and the following random intercepts: Subject nested within Testing Site. The selected mixed effect model (conditional R^2 = 0.90, marginal R^2 = 0.23) to examine whether hearing loss severity (as measured with CDTT) significantly predicted gait speed at baseline (Hypothesis 1) included the following fixed effects: CDTT x Condition, CDTT x MoCA, Age, Education, and Sex, and the following random intercepts: Subject nested within Testing Site.

Stride time. The selected mixed effect model (conditional R^2 = 0.89, marginal R^2 = 0.16) to examine whether hearing loss severity (as measured with HHIE) significantly predicted stride time at baseline (Hypothesis 1) included the following fixed effects: HHIE x Condition, HHIE x MoCA, Age, Education, and Sex, and the following random intercepts: Subject nested within Testing Site. The selected mixed effect model (conditional R^2 = 0.89, marginal R^2 = 0.25) to examine whether hearing loss severity (as measured with CDTT) significantly predicted stride time at baseline (Hypothesis 1) included the following fixed effects: CDTT x Condition, CDTT * MoCA, Age, Education, and Sex, and the following random intercepts: Subject nested within Testing Site.

Stride time variability. The selected mixed effect model (conditional R^2 = 0.37, marginal R^2 = 0.15) to examine whether hearing loss severity (as measured with HHIE) significantly predicted stride time variability at baseline (Hypothesis 1) included the following fixed effects: HHIE x Condition, MoCA x Age, Education, and Sex, and the following random intercepts: Subject nested

within Testing Site. The selected mixed effect model (conditional R^2 = 0.33, marginal R^2 = 0.13) to examine whether hearing loss severity (as measured with CDTT) significantly predicted stride time variability at baseline (Hypothesis 1) included the following fixed effects: CDTT x Condition, MoCA x Age, Education, and Sex, and the following random intercepts: Subject nested within Testing Site.

Stride length. The selected mixed effect model (conditional R^2 = 0.89, marginal R^2 = 0.20) to examine whether hearing loss severity (as measured with HHIE) significantly predicted stride length at baseline (Hypothesis 1) included the following fixed effects: HHIE x Condition, Age x Condition, MoCA, Education, and Sex, and the following random intercepts: Subject nested within Testing Site. The selected mixed effect model (conditional R^2 = 0.94, marginal R^2 = 0.19) to examine whether hearing loss severity (as measured with CDTT) significantly predicted stride length at baseline (Hypothesis 1) included the following fixed effects: CDTT x Condition, Age x Condition, MoCA Education, and Sex, and the following random intercepts: Subject nested within Testing Site.

Stride length variability. The selected mixed effect model (conditional R^2 = 0.32, marginal R^2 = 0.14) to examine whether hearing loss severity (as measured with HHIE) significantly predicted stride length variability at baseline (Hypothesis 1) included the following fixed effects: HHIE x Condition, HHIE x MoCA, Age x Condition, Education, and Sex, and the following random intercepts: Subject nested within Testing Site. The selected mixed effect model (conditional R^2 = 0.30, marginal R^2 = 0.20) to examine whether hearing loss severity (as measured with CDTT) significantly predicted stride length variability at baseline (Hypothesis 1) included the following fixed effects: CDTT x Condition, MoCA x CDTT, CDTT x Age, CDTT x Education, and Sex, and the following random intercepts: Subject nested within Testing Site.

Double support time. The selected mixed effect model (conditional R^2 = 0.85, marginal R^2 = 0.16) to examine whether hearing loss severity (as measured with HHIE) significantly predicted double support time at baseline (Hypothesis 1) included the following fixed effects: HHIE x

Condition, Age x MoCA, Education, and Sex, and the following random intercepts: Subject nested within Testing Site. The selected mixed effect model (conditional R^2 = 0.85, marginal R^2 = 0.33) to examine whether hearing loss severity (as measured with CDTT) significantly predicted double support time at baseline (Hypothesis 1) included the following fixed effects: CDTT x Condition, CDTT x MoCA, CDTT x Age, MoCA x Age, Education, and Sex, and the following random intercepts: Subject nested within Testing Site.

Training Models

Cognitive performance. The selected mixed effect model (conditional R^2 = 0.73, marginal R^2 = 0.66) to examine whether hearing loss severity (as measured with HHIE) significantly predicted degree of improvement in cognitive accuracy following training (Hypothesis 2) included the following fixed effects: HHIE x Training, HHIE x Education, HHIE x Condition, HHIE x Sex, Education x Condition, and MoCA x Age, and the following random intercepts: Subject nested within Testing Site. The selected mixed effect model (conditional R^2 = 0.70, marginal R^2 = 0.64) to examine whether hearing loss severity (as measured with CDTT) significantly predicted degree of improvement in cognitive accuracy following training (Hypothesis 2) included the following fixed effects: CDTT x Training x Time, CDTT x Sex, Age, Education, MoCA, and Condition, and the following random intercepts: Subject nested within Testing Site.

Gait speed. The selected mixed effect model (conditional R^2 = 0.83, marginal R^2 = 0.26) to examine whether hearing loss severity (as measured with HHIE) significantly predicted degree of improvement in gait speed following training (Hypothesis 2) included the following fixed effects: HHIE x Training, HHIE x Sex, Age, Education, MoCA, and Condition, and the following random intercepts: Subject nested within Testing Site. The selected mixed effect model (conditional R^2 = 0.83, marginal R^2 = 0.30) to examine whether hearing loss severity (as measured with CDTT) significantly predicted degree of improvement in gait speed following training (Hypothesis 2)

included the following fixed effects: CDTT x Training x Time, CDTT x MoCA, Age, Sex, Education, and Condition, and the following random intercepts: Subject nested within Testing Site.

Stride time. The selected mixed effect model (conditional R^2 = 0.80, marginal R^2 = 0.19) to examine whether hearing loss severity (as measured with HHIE) significantly predicted degree of improvement in stride time following training (Hypothesis 2) included the following fixed effects: HHIE x Training x Time, HHIE x Sex, Age, Education, MoCA, and Condition, and the following random intercepts: Subject nested within Testing Site. The selected mixed effect model (conditional R^2 = 0.79, marginal R^2 = 0.32) to examine whether hearing loss severity (as measured with CDTT) significantly predicted degree of improvement in stride time following training (Hypothesis 2) included the following fixed effects: CDTT x Training x Time, CDTT x Sex, CDTT x MoCA, Education x MoCA, Age, and Condition, and the following random intercepts: Subject nested within Testing Site.

Stride time variability. The selected mixed effect model (conditional R^2 = 0.37, marginal R^2 = 0.13) to examine whether hearing loss severity (as measured with HHIE) significantly predicted degree of improvement in stride time variability following training (Hypothesis 2) included the following fixed effects: HHIE x Training x Time, MoCA x Education, Age, Sex, and Condition, and the following random intercepts: Subject nested within Testing Site. The selected mixed effect model (conditional R^2 = 0.31, marginal R^2 = 0.10) to examine whether hearing loss severity (as measured with CDTT) significantly predicted degree of improvement in stride time variability following training (Hypothesis 2) included the following fixed effects: CDTT x Training x Time, CDTT x Sex, Age, MoCA, Education, and Condition, and the following random intercepts: Subject nested within Testing Site.

Stride length. The selected mixed effect model (conditional R^2 = 0.86, marginal R^2 = 0.28) to examine whether hearing loss severity (as measured with HHIE) significantly predicted degree of

improvement in stride length following training (Hypothesis 2) included the following fixed effects: HHIE x Training x Time, HHIE x Sex, Age, MoCA, Education, and Condition, and the following random intercepts: Subject nested within Testing Site. The selected mixed effect model (conditional R^2 = 0.87, marginal R^2 = 0.24) to examine whether hearing loss severity (as measured with CDTT) significantly predicted degree of improvement in stride length following training (Hypothesis 2) included the following fixed effects: CDTT x Training x Time, CDTT x Condition, Sex, Age, MoCA, and Education, and the following random intercepts: Subject nested within Testing Site.

Stride length variability. The selected mixed effect model (conditional R^2 = 0.27, marginal R^2 = 0.16) to examine whether hearing loss severity (as measured with HHIE) significantly predicted degree of improvement in stride length variability following training (Hypothesis 2) included the following fixed effects: HHIE x Training x Time, HHIE x Sex, HHIE x Age, MoCA x Age, Education, and Condition, and the following random intercepts: Subject nested within Testing Site. The selected mixed effect model (conditional R^2 = 0.21, marginal R^2 = 0.16) to examine whether hearing loss severity (as measured with CDTT) significantly predicted degree of improvement in stride length variability following training (Hypothesis 2) included the following fixed effects: CDTT x Training x Time, CDTT x Education, MoCA x Age, Sex, and Education, and the following random intercepts: Subject nested within Testing Site.

Double support time. The selected mixed effect model (conditional R^2 = 0.75, marginal R^2 = 0.27) to examine whether hearing loss severity (as measured with HHIE) significantly predicted degree of improvement in double support time following training (Hypothesis 2) included the following fixed effects: HHIE x Training x Time, HHIE x Education, HHIE x Sex, MoCA x Age, and Condition, and the following random intercepts: Subject nested within Testing Site. The selected mixed effect model (conditional R^2 = 0.74, marginal R^2 = 0.30) to examine whether hearing loss severity (as measured with CDTT) significantly predicted degree of improvement in double support

time following training (Hypothesis 2) included the following fixed effects: CDTT x Training x Time, CDTT x MoCA, Age, Sex, Education, and Condition, and the following random intercepts: Subject nested within Testing Site.

Results

Effect of Hearing Loss on Single- and Dual-Task Performance at Baseline

To address our first research question of whether dual-task performance differed across MCI participants with normal hearing or hearing loss at baseline, we first examined whether there were any differences in participant characteristics across hearing groups (Supplementary Table 1). Hearing groups were not significantly different with regards to age, MoCA scores, or the proportion of males and females. However, participants with hearing loss had significantly fewer years of education compared to those with normal hearing. Years of education was therefore included as a covariate in all further analyses. HHIE-S scores were also significantly higher in participants with hearing loss compared to normal hearing, indicating a higher degree of perceived impediment due to hearing loss.

In examining single- and dual-task gait performance across hearing groups at baseline (Research Question 1), we found a significant main effect of Task for stride time, F (1, 211) = 21.84, $p < .001$, $\eta_p^2 = .09$, stride time variability, F (1, 211) = 5.39, $p = .02$, $\eta_p^2 = .02$, stride length, F (1, 211) = 5.22, $p = .02$, $\eta_p^2 = .02$, stride length variability, F (1, 211) = 7.64, $p = .006$, $\eta_p^2 = .03$, and double support time, F (1, 211) = 18.49, $p < .001$, $\eta_p^2 = .08$, such that performance was worse under dual-task conditions compared to the single-task condition (Supplementary Table 2). We found marginally significant Hearing Ability by Task interaction effects for stride time variability, F(1, 211) = 3.55, $p = .06$, $\eta_p^2 = .02$, and stride length variability, F(1, 211) = 2.77, $p = .097$, $\eta_p^2 = .01$, such that dual-task performance was significantly worse compared to single-task performance in participants with hearing loss, but not in participants with normal hearing (Supplementary Table 2).

To better understand the marginally significant Hearing Ability by Task interaction effects for stride time variability and stride length variability, we examined the relative performance across single- and dual-task domains (i.e., dual-task costs [DTC]: single-task – dual-task/single-task*100) between hearing groups. We found a significant main effect of Hearing Ability for stride time variability DTCs, *F* (1, 154) = 5.64, I = .019, η_p^2 = .04, such that participants with hearing loss had higher DTCs (*M* = 35.4%, *SE* = 10.53), compared to participants with normal hearing (*M* = 15.0%, *SE* = 6.13).

Marginally significant Hearing Ability by Condition interactions were also found for stride time variability, *F*(3, 207) = 2.13, *p* = .098, η_p^2 = .03, and stride length variability, *F*(3, 207) = 2.31, *p* = .078, η_p^2 = .03. Post hoc analyses revealed that in participants with normal hearing, stride time variability was significantly higher when walking was paired with S7 subtractions (*M* = 3.17%, SE = .18) compared to S1 subtractions (*M* = 2.39%, *SE* = .18, *p* = .002). There were no significant differences in stride time variability across conditions in participants with hearing loss. Post hoc comparisons for stride length variability revealed that in participants with hearing loss, stride length variability was significantly higher in the semantic fluency condition (*M* = 4.27%, *SE* = .35) compared to the S1 subtraction condition (*M* = 3.04%, *SE* = .35, *p* = .02). There were no significant differences in stride length variability across conditions in participants with normal hearing. Finally, within the semantic fluency condition, stride length variability was found to be marginally higher in participants with hearing loss compared to normal hearing (*M* = 3.47%, *SE* = .21, *p* = .05).

In contrast to our hypotheses, we did not find any significant differences in cognitive accuracy across participants with normal hearing and hearing loss at baseline. We also did not find any significant differences across single-task and dual-task cognitive performance at baseline (Supplementary Table 2).

Effect of Hearing Loss on the Change in Single- and Dual-Task Performance from Pre- to Post-Training

In examining whether hearing ability moderated single- and dual-task performance from pre- to post-training (Research Question 2), we found significant Time by Hearing Group interaction effects for dual-task stride time, $F(2, 345) = 7.21$, $p = .008$, $F(2, 346) = 7.20$, $p = .007$, $\eta_p^2 = .02$, stride time variability, $F(2, 349) = 6.15$, $p = .01$, $\eta_p^2 = .02$, and double support time, $F(1, 346) = 5.64$, $p = .02$, $\eta_p^2 = .02$. Specifically, dual-task stride time ($\Delta = -50.82$ ms, $SE = 12.66$, $p < .001$), stride time variability ($\Delta = .60\%$, $SE = .23$, $p = .01$), and double support time ($\Delta = -27.49$ ms, $SE = 7.62$, $p < .001$) significantly improved in participants with hearing loss, but not in participants with normal hearing, regardless of training modality (Supplementary Figures 1-3). There was also a significant Time by Training Group by Hearing Group interaction for dual-task stride length, $F(2, 347) = 3.86$, $p = .02$, $\eta_p^2 = .02$. Post hoc pairwise analyses revealed that following Ex + CT, dual-task stride length significantly improved in participants with hearing loss ($\Delta = 7.53$ cm, $SE = 2.00$, $p < .001$), and marginally improved in participants with normal hearing ($\Delta = 2.52$ cm, $SE = 1.28$, $p = .05$).

There was no effect of hearing loss on single-task gait performance following training. We further examined DTC scores for the reported interactions to elucidate whether individual differences in single-task gait measures affected the observed patterns. We found a significant Time by Hearing Ability interaction for stride time DTCs, $F(1, 348) = 5.60$, $p = .02$, $\eta_p^2 = .02$, such that DTCs significantly decreased (i.e., improved) in participants with hearing loss ($\Delta = -1.82\%$, $SE = .89$, $p = .04$), but not in participants with normal hearing ($\Delta = 0.34\%$, $SE = 0.55$, $p = .53$). There was a marginally significant time by Hearing Ability interaction for stride time variability DTCs $F(1, 350) = 3.77$, $p = .05$, $\eta_p^2 = .01$, such that DTCs significantly decreased in hearing loss participants only ($\Delta = -29.2\%$, $SE = 11.02$, $p = .01$). Finally, we found a significant Time by Hearing Ability by Training

Group interaction for double support time DTCs, F (2, 350) = 4.00, p = .02, η_p^2 = .02, such that following the BAT + sham CT training, double support time DTCs significantly increased (i.e., worsened) in normal hearing participants (Δ = 7.07%, *SE* = 3.00, p = .01), whereas DTCs significantly decreased in participants with hearing loss (Δ = -9.96%, *SE* = 4.87, p = .04).

Regarding cognitive accuracy, there were no significant changes in both single- and dual-task performance following training, nor were there any significant differences found across hearing groups (see Supplementary Table 3 for estimated marginal means of dual-task gait and cognitive performance before and after training across hearing groups and intervention arms).

Supplementary Table 1

Means, Standard Deviations, and One-Way ANOVAs/Chi Square Tests for Participant Characteristics and Background Measures Across Hearing Groups at Baseline

	Normal Hearing $n = 56$		Hearing Loss $n = 19$		*F or* χ^2	*p*	η_p^2
	M	*SD*	*M*	*SD*			
Age (years)	72.86	7.06	76.00	5.23	3.15	.080	.041
Sex (*n*, % female)	24 (42.86)		8 (42.11)		.003	.954	
Education (years)	16.24	4.22	14.16	2.50	4.11*	.046	.053
MoCA (/30)	23.34	2.89	21.89	3.04	3.45	.067	.045
HHIE-S (/40)	5.20	6.34	17.47	9.45	40.53**	<.001	.498
CDTT SRT (SNR)	-10.36	1.30	-4.23	5.14	49.04	<.001	.490

Note. MoCA = Montreal Cognitive Assessment; HHIE-S = Hearing Handicap Inventory For The Elderly - Screening Version; CDTT SRT = Canadian Digit Triplet Test – Speech Response Threshold; SNR = Signal to noise ratio; Hearing loss was characterized by an abbreviated pure-tone audiometry assessment, wherein participants who were unable to detect a 2-kHz tone at 25 dB HL in both ears were classified as being hearing impaired; * $p < .05$, ** $p < .001$

Supplementary Table 2

Single- and Dual-Task Gait and Cognitive Performance across Hearing Groups at Baseline

	Normal Hearing (n = 56)		Hearing Loss (n = 19)		Total (n = 75)	
	ST	DT	ST	DT	ST	DT
Stride length (cm)	132 (2.40)	128 (2.27)***	126 (4.34)	131 (4.54)*	131 (2.52)	127 (2.43)***
Stride length variability (%)	3.07 (.21)	3.35 (.15)	2.66 (.35)	3.60 (.25)**	2.86 (.20)	3.48 (.14)**
Stride time (ms)	1091 (17.4)	1166 (16.1)***	1072 (29.7)	1145 (27.6)***	1081 (16.5)	1156 (15.5)***
Stride time variability (%)	2.75 (.18)	2.78 (.13)	2.30 (.30)	2.98 (.21)*	2.52 (.17)	2.88 (.12)*
Double support time (ms)	323 (9.38)	348 (8.48)***	313 (16.00)	353 (14.57)***	318 (9.07)	350 (8.20)***
Cognitive accuracy (correct/time)	.49 (.03)	.56 (.03)	.57 (.06)	.61 (.06)	.53 (.03)	.58 (.03)

Note. ST = single-task; DT = dual-task; Hearing loss was characterized by an abbreviated pure-tone audiometry assessment, wherein participants who were unable to detect a 2-kHz tone at 25 dB HL in both ears were classified as being hearing impaired; Dual-task gait performance was aggregated across the serial 1 subtractions, serial 7 subtractions, and semantic fluency conditions; Cognitive performance was aggregated across the serial 7 subtraction and semantic fluency tasks for both the single- and dual-task conditions; Asterisks indicate significant differences between single-task and dual-task; *** $p < .001$; ** $p < .01$; * $p < .05$.

Supplementary Table 3

Estimated Marginal Means and Standard Errors of Dual-Task Performance Before and After Training Across Hearing Groups and Intervention Arms

Measure	Ex + CT *n* = 32				Ex + sham CT *n* = 31				BAT + sham CT *n* = 12			
	Normal Hearing n = 23		Hearing Loss *n* = 9		Normal Hearing *n* = 24		Hearing Loss *n* = 7		Normal Hearing *n* = 9		Hearing Loss *n* = 3	
	T1	T2	T1	T2	T1	T2	T1	T2	T1	T2	T1	T2
Gait speed (cm/s)	112 (4.74)	119 (4.74)*	118 (6.62)	124 (6.62)*	107 (4.72)	112 (4.71)*	113 (8.07)	117 (8.07)*	108 (7.52)	108 (7.47)	113 (9.03)	113 (9.00)
Stride time (ms)	1160 (26.9)	1134 (26.9)*	1126 (40.4)	1078 (40.4)*	1174 (25.8)	1171 (25.8)	1154 (50.5)	1117 (50.5)*	1140 (44.0)	1163 (43.6)	1209 (70.2)	1142 (70.2)*
Stride time variability (CoV%)	2.74 (.22)	2.50 (.22)	3.11 (.33)	2.51 (.33)*	2.79 (.21)	2.95 (.21)	2.61 (.41)	2.59 (.41)	2.84 (.37)	3.19 (.35)	3.49 (.57)	2.33 (.57)*
Stride length (cm)	129 (3.75)	132 (3.74)	123 (5.62)	130 (5.62)*	123 (3.57)	126 (3.57)*	121 (7.76)	119 (7.76)	127 (6.09)	128 (6.05)	123 (9.81)	123 (9.81)
Stride length variability (CoV%)	3.24 (.25)	3.05 (.24)	4.00 (.37)	3.56 (.37)	3.56 (.24)	3.63 (.24)	3.12 (.45)	3.15 (.45)	3.29 (.43)	3.85 (.40)	3.56 (.64)	3.13 (.64)
Double support time (ms)	341 (13.7)	327 (13.6)*	331 (20.5)	311 (20.5)*	356 (13.1)	353 (13.1)	356 (25.6)	337 (25.6)	345 (22.9)	352 (22.6)	393 (35.5)	352 (35.5)*
Double support time variability (CoV)	7.37 (.51)	5.65 (.50)*	6.16 (.76)	5.02 (.76)	6.38 (.49)	6.49 (.49)	5.36 (1.02)	5.82 (1.02)	6.74 (.84)	6.91 (.82)	6.50 (1.33)	4.71 (1.33)
Cognitive accuracy (number correct/ time)	.79 (.04)	.85 (.04)	.85 (.07)	.81 (.07)	.75 (.04)	.83 (.04)	.86 (.12)	.75 (.12)	.69 (.07)	.73 (.69)	.93 (.12)	.98 (.12)

Note. T1 = Time 1 (baseline); T2 = Time 2 (post-training); Standard error in brackets; Hearing loss was characterized by an abbreviated pure-tone audiometry assessment, wherein participants who were unable to detect a 2-kHz tone at 25 dB HL in both ears were classified as being hearing impaired; Performance in each domain is the aggregated score across the serial 1 subtractions, serial 7 subtractions, and semantic fluency conditions; cognitive accuracy scores were calculated by dividing the total number of correct items divided by the time to complete the walking trial; * $p < .05$ between pre- and post-training.

Supplementary Figure 1

Dual-Task Stride Time Before and After Training Across Participants with Hearing Loss and Normal Hearing

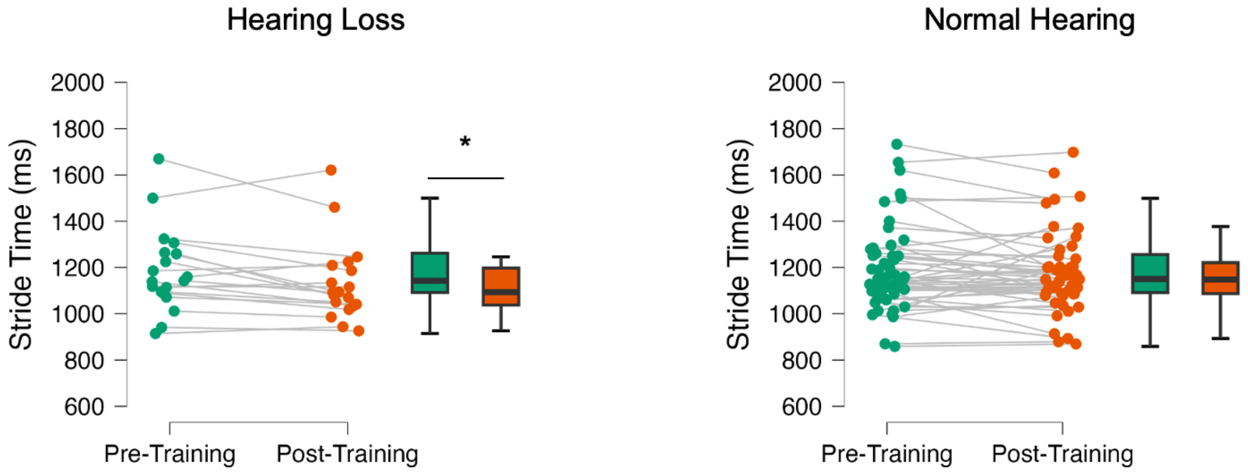

Note. Hearing loss was characterized by an abbreviated pure-tone audiometry assessment, wherein participants who were unable to detect a 2-kHz tone at 25 dB HL in both ears were classified as being hearing impaired; Each circle represents a participant, with the connecting line depicting the individual change scores from pre- to post-training; Dual-task performance was aggregated across the serial 1 subtractions, serial 7 subtractions, and semantic fluency conditions. Training was aggregated across the three intervention arms (i.e., aerobic-resistance exercise (Ex) with cognitive training (CT), Ex with sham CT, and balance and toning with sham CT); * $p < .01$.

Supplementary Figure 2

Dual-Task Stride Time Variability Before and After Training Across Participants with Hearing Loss and Normal Hearing

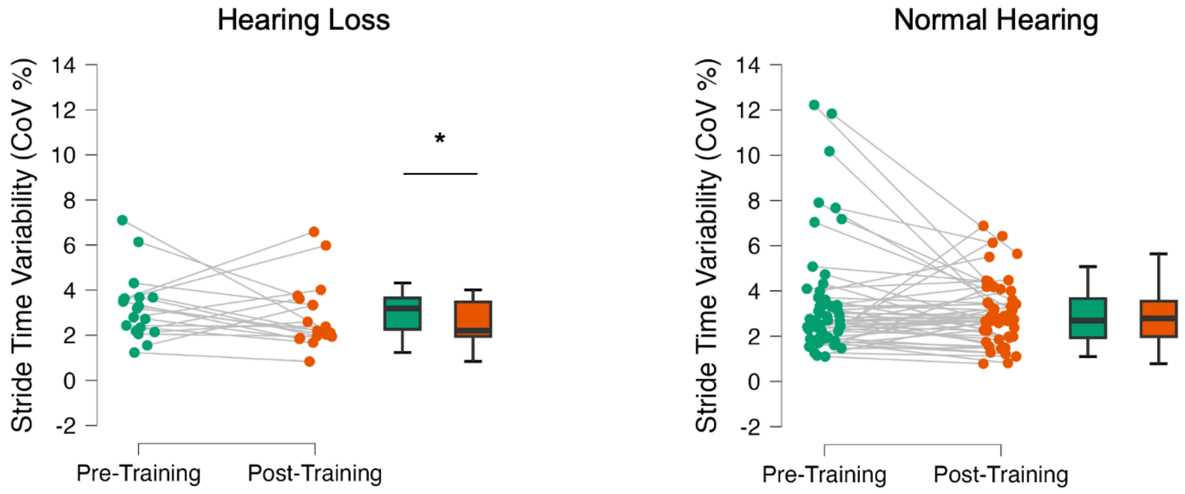

Note. Hearing loss was characterized by an abbreviated pure-tone audiometry assessment, wherein participants who were unable to detect a 2-kHz tone at 25 dB HL in both ears were classified as being hearing impaired; Each circle represents a participant, with the connecting line depicting the individual change scores from pre- to post-training; Dual-task performance was aggregated across the serial 1 subtractions, serial 7 subtractions, and semantic fluency conditions. Training was aggregated across the three intervention arms (i.e., aerobic-resistance exercise (Ex) with cognitive training (CT), Ex with sham CT, and balance and toning with sham CT); * $p < .05$.

Supplementary Figure 3

Dual-Task Double Support Time Before and After Training Across Participants with Hearing Loss and Normal Hearing

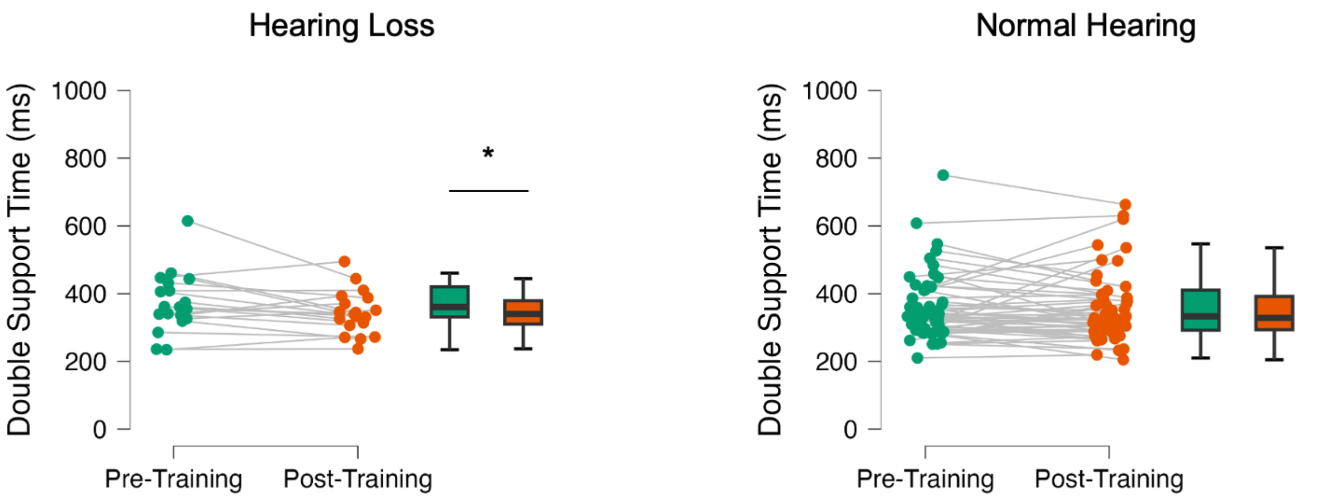

Note. Hearing loss was characterized by an abbreviated pure-tone audiometry assessment, wherein participants who were unable to detect a 2-kHz tone at 25 dB HL in both ears were classified as being hearing impaired; Each circle represents a participant, with the connecting line depicting the individual change scores from pre- to post-training; Dual-task performance was aggregated across the serial 1 subtractions, serial 7 subtractions, and semantic fluency conditions. Training was aggregated across the three intervention arms (i.e., aerobic-resistance exercise (Ex) with cognitive training (CT), Ex with sham CT, and balance and toning with sham CT); * $p < .05$.
